# High chemoselectivity in the phenol synthesis

**DOI:** 10.3762/bjoc.7.90

**Published:** 2011-06-10

**Authors:** Matthias Rudolph, Melissa Q McCreery, Wolfgang Frey, A Stephen K Hashmi

**Affiliations:** 1 Organisch-Chemisches Institut, Ruprecht-Karls-Universität Heidelberg, Im Neuenheimer Feld 270, 69120 Heidelberg, Germany; 2 Institut für Organische Chemie, Universität Stuttgart, Pfaffenwaldring 55, 70569 Stuttgart, Germany

**Keywords:** alcohols, alkenes, alkynes, furans, gold, ketones

## Abstract

Efforts to trap early intermediates of the gold-catalyzed phenol synthesis failed. Neither inter- nor intramolecularly offered vinyl groups, ketones or alcohols were able to intercept the gold carbenoid species. This indicates that the competing steps of the gold-catalyzed phenol synthesis are much faster than the steps of the interception reaction. In the latter the barrier of activation is higher. At the same time this explains the high tolerance of this very efficient and general reaction towards functional groups.

## Introduction

**Scheme 1:**
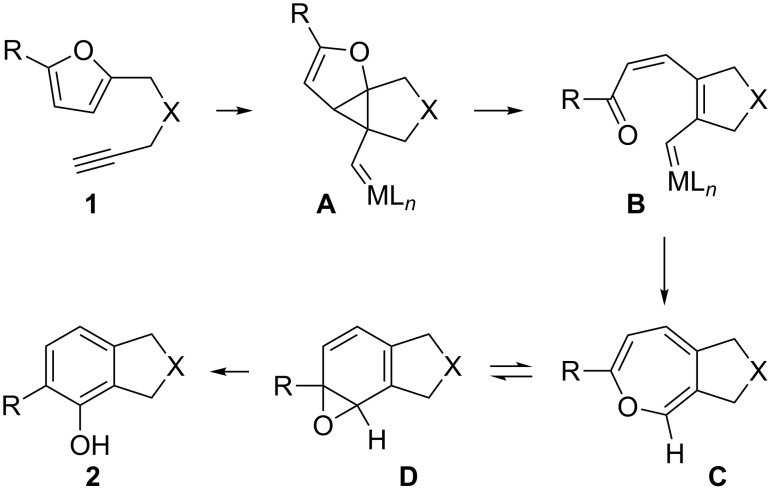
Mechanism of the furan–yne reaction.

As documented in numerous reviews [1–10], over the last eleven years homogeneous gold catalysis has emerged from early examples [11–12] which documented its potential for organic synthesis of even complex molecules to an established tool in preparative organic chemistry [13–14]. One of these early examples is the gold-catalyzed phenol synthesis [12] in which the furan-ynes **1** used as substrates represent the first ene–yne-type compounds ever used in gold catalysis. While many investigations in the field focused on methodology, mechanistic research was much less widespread [2–3,15]. The gold-catalyzed ene–yne cycloisomerization reactions are, mechanistically, very complex reactions [16–18], and the furan–yne cycloisomerization is no exception. For the latter reaction arene oxides **D** [19] and oxepines **C** [20] could be detected as intermediates, and these could even be trapped by Diels–Alder reactions. In addition, labelling studies were carried out and the electronic influence of substituents was investigated [21]. Computational studies as well as side-products produced in the reaction pointed towards intermediates **A** and **B** (Scheme 1) [22–25]. Moreover, interesting new pathways were opened when ynamides and alkynyl ether substrates were employed: Here **A** is also a possible intermediate along these pathways [25].

Since direct experimental evidence existed only for **C** and **D**, we intended to intercept the postulated carbenoid intermediates **A** or **B**. Apart from intermolecular trapping [26–33], intramolecular trapping of such carbenoids has also been reported [34]. One option would be to offer a competing carbonyl group, to produce a carbonyl ylide, which could then undergo a 1,3-dipolar cycloaddition [35]. The second option would be a classical cyclopropanation of an olefin. A third option would be trapping of intermediate **A** with an intramolecular hydroxy nucleophile [36]. Here we report our observations when trying to apply these principles to intermediates of type **A** or **B**.

## Results and Discussion

### Intermolecular olefinic trapping reagents

**Scheme 2:**
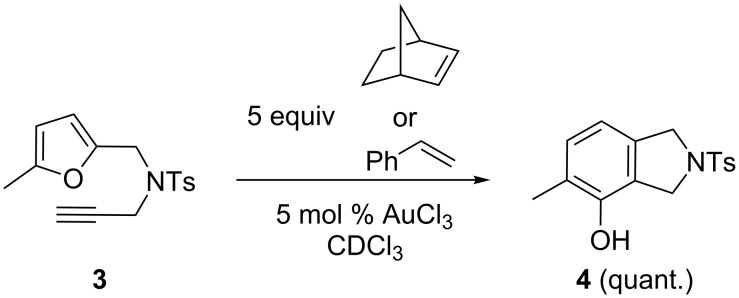
Efforts for intermolecular trapping with olefins failed.

We started with the simplest experiments, namely the intermolecular trapping of the gold carbenoid intermediates. When **3** was reacted in the presence of an activated olefin, such as norbornene or styrene, phenol **4** was formed exclusively in essentially quantitative yield, no other products could be detected (Scheme 2).

**Scheme 3:**
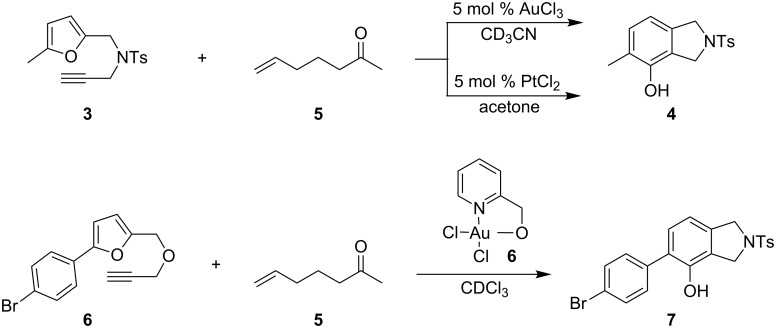
Efforts for intermolecular trapping with ketones failed.

Experiments with a competing carbonyl group (competing with the carbonyl group in intermediate **B**) were also unsuccessful. Ketone **5** [37], prepared by the addition of methyllithium to commercially available hex-5-enoic acid, was used as an external carbonyl group. Reaction with both tosylamide **3** and ether **6** always delivered the phenolic products **4** or **7**, respectively (Scheme 3). The same result was obtained when PtCl_2_ was used as the catalyst for the conversion of **3**.

### Intramolecular olefinic trapping reagents

**Scheme 4:**
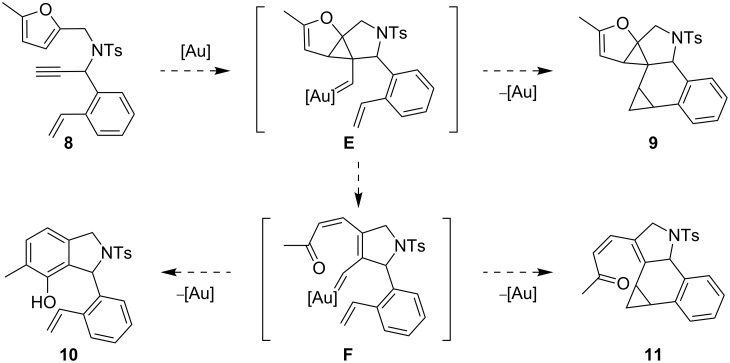
Potential products of an intramolecular trapping experiment with substrate **8**.

The next step was to offer the styrene unit in an intramolecular manner. Substrate **8** could potentially undergo three different modes of reaction (Scheme 4). After the initial step, the intermediate **E** would be produced (analogous to **A**). Cyclopropanation of the styrene subunit by the cyclopropyl carbenoid would deliver **9**. If **E** rearranged to the vinylcarbenoid **F**, the two competing reactions would be the formation of the phenol **10** and cyclopropanation to form **11**.

**Scheme 5:**
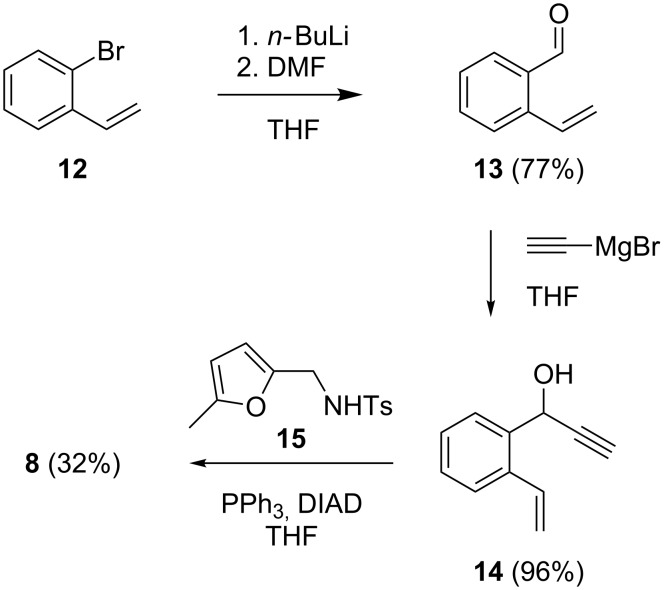
Synthesis of the substrate **8**.

The synthesis of **8** was possible by a short route (Scheme 5). Starting from the commercially available 2-bromostyrene (**12**), a halogen–metal exchange and subsequent formylation according to a procedure of Fukumoto et al. [38] gave **13**. Addition of ethynylmagnesium bromide to **13** led to **14**, which reacted with furan **15** [40] under Mitsunobu conditions [39] to afford **8**. While the yields were good for the first two steps of the reaction sequence, the yield of the last step was only 32%.

**Scheme 6:**
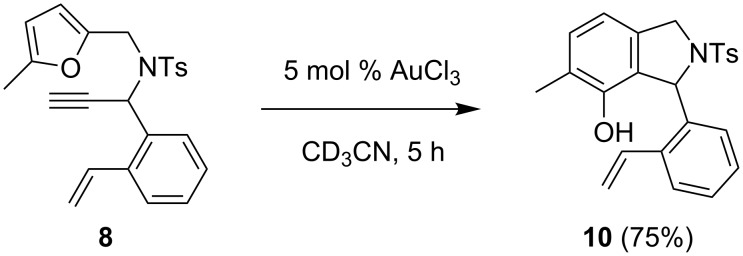
With substrate **8** the product of the phenol synthesis was exclusively obtained.

**Figure 1:**
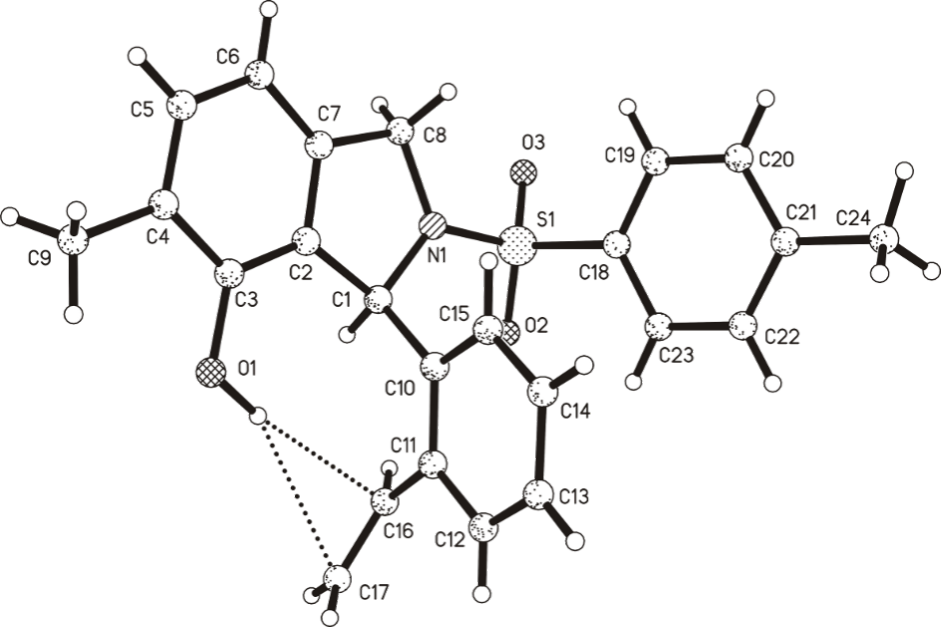
Solid-state molecular structure of **10**.

With AuCl_3_ the phenol **10** was formed exclusively (Scheme 6). The structure was unambiguously confirmed by X-ray crystal structure analysis (Figure 1). It shows an interesting hydrogen bond-like interaction of the phenolic hydroxy group and the alkene unit. After changing the solvent from acetonitrile to CDCl_3_, and the gold(I) catalyst to [Mes_3_PAu]NTf_2_ [41], only **10** was again observed. Thus, neither of the two oxidation states of the gold catalyst gave any product derived from the intercepted intermediate (the solvent was changed to CDCl_3_ since the activity of gold(I) is significantly reduced by MeCN).

### Intramolecular ketone as potential trapping reagent

**Scheme 7:**
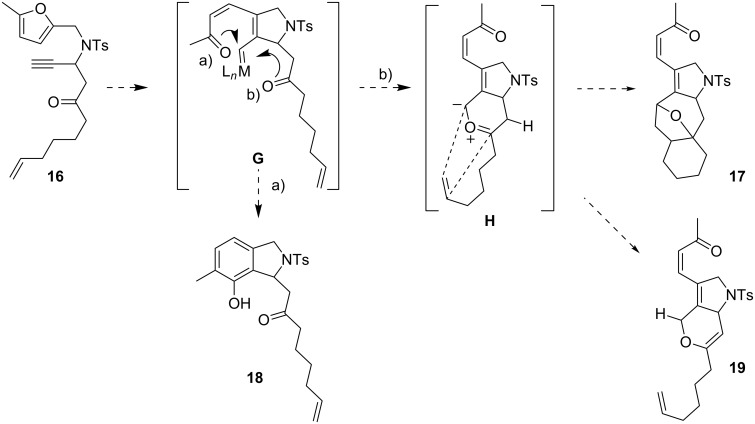
Potential products of an intramolecular trapping experiment with substrate **16**.

Next we decided to use a carbonyl group as the competing unit. The intermediate **G**, formed from substrate **16**, would offer the option of competition of the phenol synthesis (Scheme 7, pathway a) to yield **18,** and reaction with the second carbonyl group (Scheme 7, pathway b). The latter would form intermediate **H**, which could then either afford product **17** via intramolecular 1,3-dipolar cycloaddition with the olefin, or could form the diene **19** by proton migration.

**Scheme 8:**
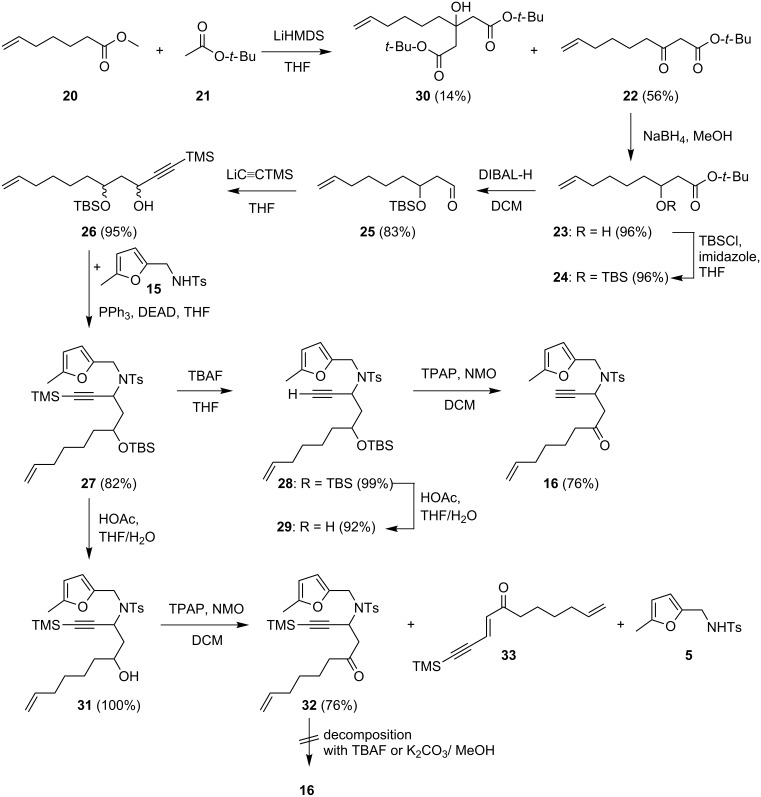
Synthesis of the substrate **16**.

The synthesis of **16** was only possible by a 9-step sequence (Scheme 8). The starting point was a Claisen condensation of ester **20** and *tert*-butyl acetate (**21**) in the presence of lithium hexamethyldisilazide as the base. Ketoester **22** was obtained in 56% yield, however, the two-fold addition of **21** could not be suppressed completely and 14% of the corresponding tertiary alcohol **30** was also obtained. Reduction of the ketone **22** with sodium borohydride and protection of the alcohol **23** with *tert*-butyldimethylsilylchloride delivered **24** in excellent yield. Reduction of the ester group with diisobutylaluminiumhydride gave aldehyde **25**. The addition of lithiated trimethylsilylacetylene provided the propargylic alcohol **26** and reaction with **15** under Mitsunobu conditions yielded **27**. Deprotection of the alkyne **2**7 and the silyl ether **28**, followed by the oxidation of the resulting alcohol **29** finally led to **16**. It was not possible to remove both silyl groups simultaneously with TBAF, longer reaction times which would be necessary for the deprotection of the hydroxy group led to decomposition of the substrate. At 0 °C and with a very short reaction time, the alkyne was deprotected selectively. Selective deprotection of the alcohol was then possible with a mixture of acetic acid/water/THF. Another route, in which the alcohol function was deprotected first, then oxidized, followed by removal of the trimethylsilyl group from the alkyne also failed. Thus treatment of **27** with acetic acid in aqueous THF gave the desired alcohol **31**in quantitative yield. However, whilst Ley oxidation [42] on the small-scale delivered ketone **32** in yields of up to 80%, on a larger scale the yield of **32** dropped dramatically to 28% and was accompanied by two side-products, **33** and **5**. The latter are formed by an elimination reaction of the amide in **32**. Furthermore, it was not possible to deprotect ketone **32** due to rapid decomposition.

**Figure 2:**
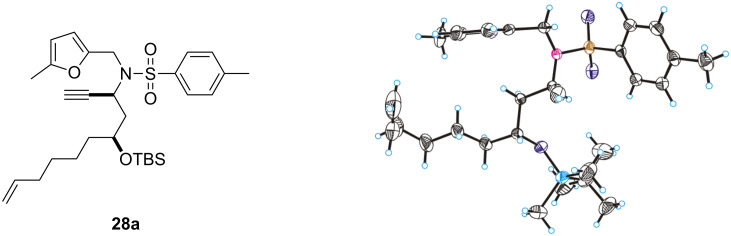
Solid-state molecular structure of **28a**.

One of the diastereoisomers of **28** was identified as the *anti*-product **28a** by an X-ray crystal structure analysis (Figure 2).

**Scheme 9:**
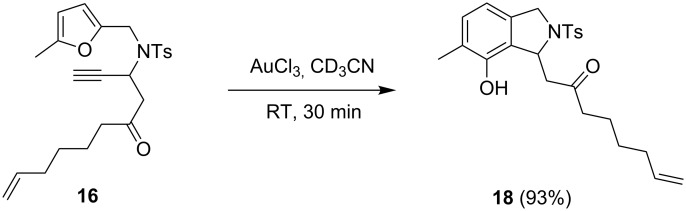
With substrate **16** the product of the phenol synthesis is obtained exclusively.

The conversion of **16** with 5 mol % AuCl_3_ proceeded fast and gave exclusively phenol **18**. No other products could be detected (Scheme 9).

**Figure 3:**
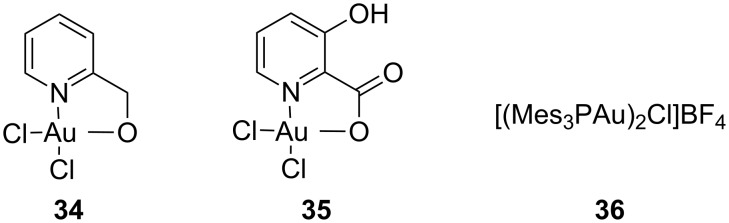
Catalysts **34**, **35** and **36**.

The two gold(III) complexes **34** [43] and **35** [37] as well as the dinuclear gold(I) complex **36** [44] gave the same result (Figure 3). When the catalyst was changed to platinum(II) chloride in acetone, a complex mixture of inseparable products was obtained.

**Figure 4:**
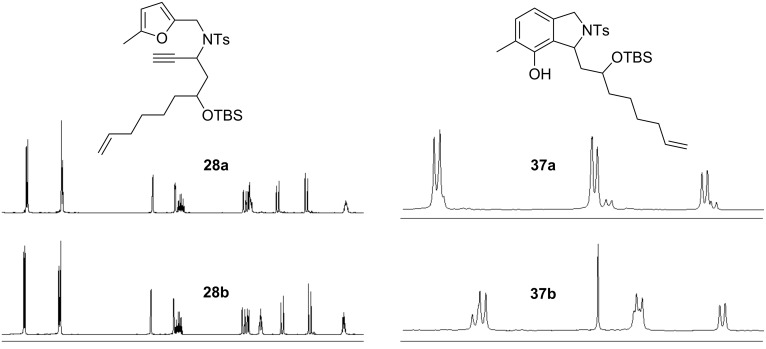
^1^H NMR spectra of the separated diastereoisomers of the substrates for catalysis **28** (left) and of the products **37** (right, the small signals are due to the deprotected compounds **38**).

**Figure 5:**
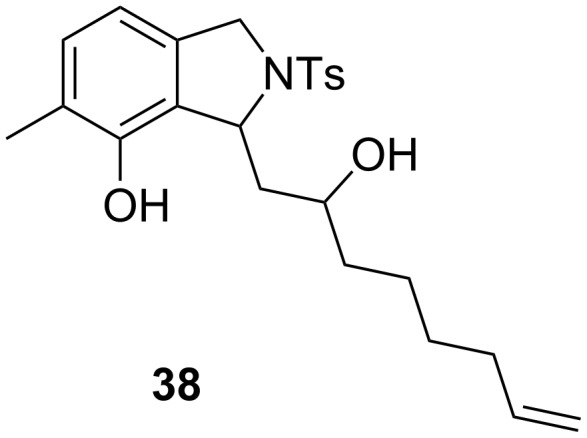
Structure of the desilylation product **38**.

Since the two diastereoisomers **28a** and **28b** with the propargylic stereocenters were separable, we investigated the gold-catalyzed conversion of the pure isomers. From the NMR spectra taken during the conversion (Figure 4), it could be clearly seen that no epimerization of the propargylic position occurred. In addition to the selective transformation to the phenols **37a** and **37b** as the main reaction products, partial removal of the TBS group was observed (**38**, Figure 5).

### Intramolecular alcohol as potential trapping reagent

**Scheme 10:**
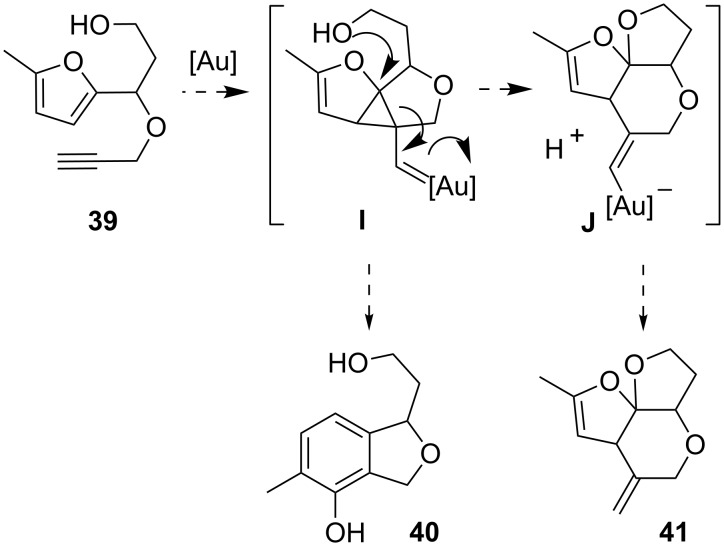
Potential products of an intramolecular trapping experiment with substrate **39**.

For the interception of intermediate **A** we also considered the option of an intramolecular hydroxy nucleophile, compound **39** (Scheme 10) would represent this type of substrate. The intermediate **I** would be an analogue of **A**. Instead of the phenol synthesis to yield **40**, an intramolecular nucleophilic attack at the activated three-membered ring could form intermediate **J**, which, after protodeauration, would provide ketal **41**.

**Scheme 11:**
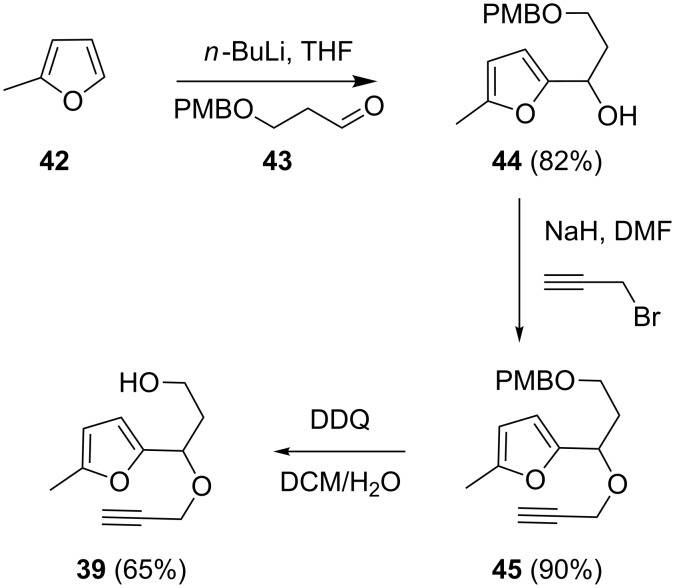
Synthesis of the substrate **39**.

The synthesis of **39** was readily accomplished by the addition of lithiated sylvan **42** to the PMB-protected aldehyde **43** (Scheme 11) [45]. The resulting furfuryl alcohol **44** was then propargylated to give **45**. The deprotection was however, problematic. Treatment of the latter with cerium ammonium nitrate led to decomposition. Only with DDQ was the desired alcohol **39** obtained in moderate yield.

**Scheme 12:**
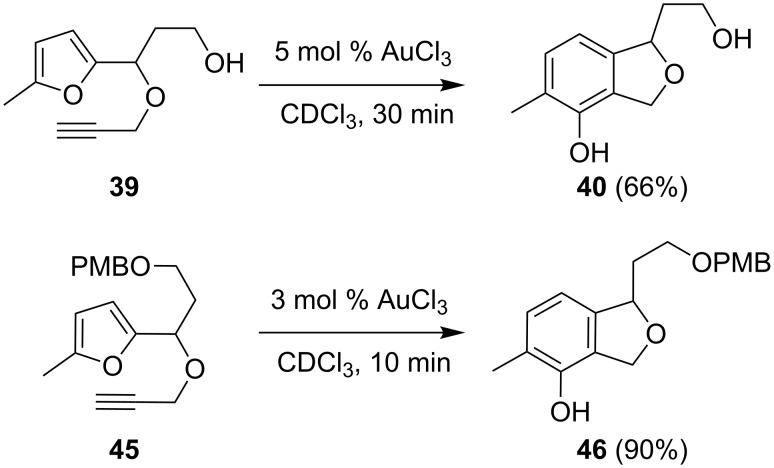
With substrate **39** and **45** exclusively the product of the phenol synthesis is obtained.

The conversion of **39**, catalyzed by AuCl_3_ in CDCl_3_, again only produced the expected phenol **40** (Scheme 12). Not unexpectedly, the PMB-protected alcohol **45** was similarly converted to **46**. PtCl_2_ did not lead to a change in selectivity.

## Conclusion

The complete failure of both the inter- and the intramolecular trapping experiments shows that the gold-catalyzed phenol synthesis follows a reaction pathway low in energy. These observations also nicely explain the high functional group tolerance, for example, towards olefins and alcohols.

## Supporting Information

File 1 Experimental details and characterization data of synthesized compounds.
