# Supplementary material for: High chemoselectivity in the phenol synthesis
Source: Beilstein J Org Chem. 2011 Jun 10;7:794–801. doi: 10.3762/bjoc.7.90 (PMC3135103; doi:10.3762/bjoc.7.90)
Supplement: File 1 — Experimental details and characterization data of synthesized compounds. [file Beilstein_J_Org_Chem-07-794-s001.pdf]

Supporting Information

for

High chemoselectivity in the phenol synthesis

Matthias Rudolph[1], Melissa Q. McCreery[2], Wolfgang Frey[2] and

A. Stephen K. Hashmi*[1,2]

Address: [1]Organisch-Chemisches Institut, Ruprecht-Karls-Universität Heidelberg, Im Neuenheimer Feld 270, 69120 Heidelberg, Germany and [2]Institut für Organische Chemie, Universität Stuttgart, Pfaffenwaldring 55, 70569 Stuttgart, Germany

Email: A. Stephen K. Hashmi - hashmi@hashmi.de

* Corresponding author

Experimental details and characterization data of synthesized compounds

General methods: Chemicals (Aldrich, Fluka, Lancaster, and Merck) were used without further purification. Diethyl ether and tetrahydrofuran were dried over sodium. NMR spectra were recorded on Bruker ARX500, ARX300 and AMX250 spectrometers. Chemical shifts were referenced to residual solvent protons. Signal multiplicity was as follows: s (singlet), d (doublet), t (triplet), q (quartet), m (multiplet). ^{13}C assignment was achieved via DEPT90 and DEPT135 spectra. MS spectra were recorded on a Finnigan MAT 90 a Varian 711 or a micrOTOF-Q spektometer. IR spectra were recorded on a Bruker Vector 22.

1-(2-Vinylphenyl)-prop-2-yn-1-ol (**14**)

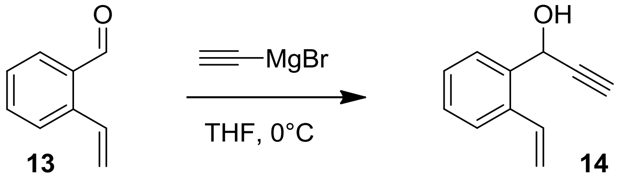

Ethynylmagnesium bromide (27.2 ml,13.6 mmol, 0.5 M solution in THF) was added at 0 °C to a solution of 1.20 g (9.08 mmol) 2-vinylbenzaldehyde (**13**) in 20 ml THF. After 15 min, NH_4Cl-solution was added and the mixture extracted three times with 20 ml Et_2O. The combined organic layers were dried over $MgSO_4$, filtered and the solvent was removed under reduced pressure. After purification by column chromatography on silica (SiO_2, PE:EA, 20:1), **14 (**1.39 g, 8.76 mmol, 96%) was obtained as a yellow solid.

Mp.: 32 °C; R_f (PE:EA, 15:1) = 0.08; IR (neat): $\tilde{\nu}$ = 3294 cm^{-1}, 3239, 1478, 1420, 1334, 1265, 1206, 1180, 1100, 1031, 987, 944, 914, 772, 613; ^1H NMR ($CDCl_3$, 300 MHz): δ = 2.25 (d, ^3J = 4.9 Hz, 1 H), 2.69 (d, ^4J = 2.2 Hz, 1 H), 5.40 (dd, ^3J = 11.0 Hz,

^2J = 1.3 Hz, 1 H), 5.70 (dd, ^3J = 17.3 Hz, ^2J = 1.3 Hz, 1 H), 5.69-5.71 (m, 1 H), 7.19 (dd, ^3J = 17.3 Hz, ^3J = 11.0 Hz, 1 H), 7.31-7.35 (m, 2 H), 7.51-7.54 (m, 1 H), 7.69-7.72 (m, 1 H); ^{13}C NMR ($CDCl_3$, 126 MHz): δ = 62.17 (d), 75.28 (d), 83.14 (s), 117.37 (t), 126.59 (d), 126.82 (d), 128.07 (d), 128.89 (d), 133.73 (d), 136.53 (s), 136.55 (s). $C_{11}H_{10}O$ (158.20): calcd. C 83.51, H 6.37; found C 83.10, H 6.43; MS (EI(+)): *m/z* (%): 158 (32) $[M]^+$, 129 (100), 115 (13), 91 (15), 51 (11); HRMS (EI (+)): $C_{11}H_{10}O$: calcd 158.0732; found 158.0731.

N-[1-(2-Ethenylphenyl)prop-2-yn-1-yl]-4-methyl-*N*-[(5-methylfuran-2-yl)methyl]benzenesulfonamide (**8**)

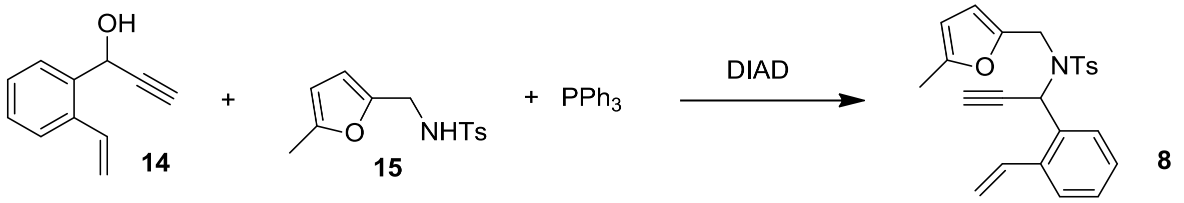

Compound **14 (**516 mg,3.26 mmol), 4-methyl-*N*-(5-methylfuran-2-ylmethyl)benzenesulfonamide (1.04 g, 3.91 mmol), PPh_3 (1.11 g, 4.24 mmol) and DIAD (840 µl, 4.24 mmol) in10 ml THF were reacted for 16 h. After purification by column chromatography on silica (SiO_2, PE:EA, 25:1), **8** (419 mg, 1.03 mmol, 32%) was obtained as a yellow solid.

Mp.: 92 °C; R_f (PE:EA, 5:1) = 0.33; IR (neat): $\tilde{\nu}$ = 3383 cm^{-1}, 2922, 1598, 1564, 1481, 1348, 1221, 1159, 1090, 1020, 945, 891, 667, 633; ^{1}H NMR ($CDCl_3$, 300 MHz): δ = 1.96 (s, 3 H), 2.31 (d, ^4J = 2.4 Hz, 1 H), 2.43 (s, 3 H), 4.13 (d, ^2J = 18.8 Hz, 1 H), 4.18 (d, ^2J = 18.8 Hz, 1 H), 5.36 (dd, ^3J = 11.0 Hz, ^2J = 1.5 Hz, 1 H), 5.55-5.58 (m, 2

H), 5.57 (dd, ^3J = 17.1 Hz, ^2J = 1.5 Hz, 1 H), 6.31 (d, ^4J = 2.4 Hz, 1 H), 7.19-7.29 (m, 4 H), 7.28 (dd, ^3J = 17.1 Hz, ^3J = 11.0 Hz, 1 H), 7.41 (dd, ^3J = 7.5 Hz, ^4J = 1.6 Hz, 1 H), 7.70 (dd, ^3J = 7.5 Hz, ^4J = 1.6 Hz, 1 H), 7.83 (d, ^3J = 8.3 Hz, 2 H); ^{13}C NMR ($CDCl_3$, 126 MHz): δ = 13.94 (q), 22.20 (q), 42.84 (t), 52.71 (d), 79.12 (d), 79.80 (s), 107.63 (d), 110.89 (d), 117.61 (t), 127.52 (d), 129.13 (d), 129.98 (d, 2 C), 130.64 (d), 130.95 (d, 2 C), 131.03 (d), 133.41 (s), 135.45 (d), 137.49 (s), 139.18 (s), 145.89 (s), 149.89 (s), 152.64 (s); MS (EI(+)): *m/z* (%): 405 $[M]^+$, 264 (21), 250 (100), 222 (25), 206 (10), 179 (11), 155 (18), 141 (34), 115 (31), 95 (77), 91 (40); HRMS (ESI (+)): $C_{24}H_{23}NNaO_3S$: calcd. 428.1219; found 428.1316.

3-(2-Ethenylphenyl)-5-methyl-2-[(4-methylphenyl)sulfonyl]-2,3-dihydro-1*H*-isoindol-4-ol (**10**)

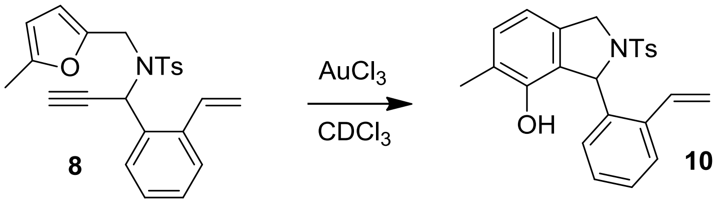

Compound **8** (50.0 mg, 123 µmol) and $AuCl_3$ (1.87 mg, 6.15 µmol) were reacted in CD_3CN for 5 h. After purification by column chromatography on silica (SiO_2, PE:EA, 10:1), **10** (37.7 mg, 930 µmol, 75%) was obtained as a colorless solid.

Mp.: 149 °C; R_f (PE:EA, 10:1) = 0.10; IR (neat): $\tilde{\nu}$ = 3543 cm^{-1}, 1598, 1498, 1462, 1340, 1157, 1105, 1076, 1040, 1005, 812, 765, 713, 662, 569; ^{1}H NMR ($CDCl_3$, 300 MHz): δ = 2.07 (s, 3 H), 2.35 (s, 3 H), 4.30 (s, 1 H), 4.78-4.89 (m, 2 H), 5.39 (dd, ^3J =

10.9 Hz, ^2J = 1.4 Hz, 1 H), 5.58 (dd, ^3J = 17.3 Hz, ^2J = 1.4 Hz, 1 H), 6.27 (s, 1 H), 6.71 (d, ^3J = 7.6 Hz, 1 H), 7.02 (m, 2 H), 7.09-7.14 (m, 3 H), 7.22-7.32 (m, 2H), 7.40 (d, ^3J = 7.8 Hz, 1 H), 7.45 (d, ^3J = 8.2 Hz, 2 H). ^{13}C NMR ($CDCl_3$, 126 MHz): δ = 15.21 (q), 21.49 (q), 54.22 (t), 63.79 (d), 114.26 (d), 118.51 (t), 123.97 (s), 126.54 (s), 127.27 (d, 2 C), 127.36 (d), 128.60 (d), 128.61 (d), 128.87 (d), 129.41 (d, 2 C), 131.46 (d), 134.51 (s), 134.64 (d), 135.71 (s), 137.17 (s), 137.26 (s), 143.40 (s), 149.03 (s); MS (EI(+)): *m/z* (%): 405 $[M]^+$ (13), 250 (100), 235 (12), 91 (14); HRMS (EI (+)): $C_{24}H_{23}NO_3S$: calcd. 405.1399; found 405.1397.

tert-Butyl-3-oxonon-8-enoate (**22**) and di-*tert*-butyl-3-hex-5-en-1-yl-3-hydroxypentandioate (**30**)

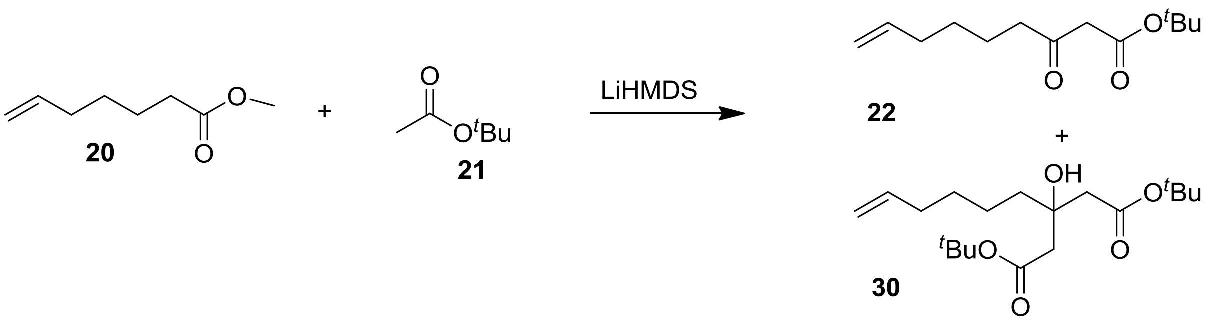

LiHMDS ((76.3 ml, 76.3 mmol, 1M solution in hexane) was added to 80 ml THF at –78 °C. *tert*-Butyl acetate (8.47 g, 72.9 mmol) was then added. After 1.5 h, methyl hept-6-enoate (4.50 g, 34.7 mmol) dissolved in 10 ml THF was added and the solution stirred at –78 °C for 1h. After the removal of the cooling bath the reaction was stirred for an additional 1.5 h. Then 100 ml 10% HCl and 50 ml Et_2O were added and the organic phase was washed 3 times with 40 ml saturated NH_4Cl solution. The

aqueous phases were extracted 3 times with 40 ml Et_2O. The combined organic layers were dried over $MgSO_4$, filtered and the solvent was removed under reduced pressure. After purification by column chromatography on silica, compound **22** (4.36 g, 19.3 mmol, 56 %) and compound **30** (1.62 g, 4.73 mmol, 14 %) were obtained as a colorless oils.

22: R_f (PE:EA, 20:1) = 0.19; IR (Film): $\tilde{\nu}$ = 3076 cm^{-1}, 2978, 2932, 2359, 1737, 1715, 1641, 1369, 1319, 1252,.1149; ^{1}H NMR ($CDCl_3$, 500 MHz): δ = 1.36-1.43 (m, 2 H), 1.57-1.63 (s, 9 H), 1.57–1.63 (m, 2 H), 2.03-2.07 (m, 2 H), 2.52 (t, ^3J = 7.3 Hz, 2 H), 3.33 (s, 2 H), 4.93-5.02 (m, 2 H), 5.74-5.82 (m, 1 H);^{13}C NMR ($CDCl_3$, 126 MHz): δ = 22.89 (t), 27.96 (q, 3 C), 28.32 (t), 33.43 (t), 42.69 (t), 50.65 (t), 81.86 (s), 114.69 (t), 138.35 (d), 166.47 (s), 203.18 (s). MS (EI(+)): m/z (%): 226 (0.2) $[M]^+$, 170 (11), 153 (14), 115 (21), 68 (21), 57 (100), 41 (33); $C_{13}H_{22}O_3$ (226.31.): calcd. C 68.99, H 9.80; found C 69.02, H 9.87.

30: R_f (PE:EA, 20:1) = 0.16; IR (Film): $\tilde{\nu}$ = 2978 cm^{-1}, 2937, 1726, 1393, 1368, 1255, 1153. ^{1}H NMR ($CDCl_3$, 500 MHz): δ = 1.31-1.43 (m, 4 H), 1.46 (s, 18 H), 1.57–1.60 (m, 2 H), 2.04-2.08 (m, 2 H), 2.49 (d, ^2J = 15.2 Hz, 2 H), 2.57 (d, ^2J = 15.2 Hz, 2 H), 4.21 (s, 1 H), 4.92-5.01 (m, 2 H), 5.76-5.84 (m, 1 H);^{13}C NMR ($CDCl_3$, 126 MHz): δ = 22.88 (t), 28.09 (q, 6 C), 29.31 (t), 33.70 (t), 39.61 (t), 44.46 (t, 2 C), 71.84 (s), 81.11 (s, 2 C), 114.37 (t), 138.81 (d), 171.40 (s, 2 C); MS (CI(+)): *m/z* (%): 343 (100) $[MH]^+$, 287 (29), 231 (30), 171 (19), 147 (16), 57 (16). $C_{19}H_{34}O_5$ (342.47): calcd. 66.63, H 10.01; found C 66.31, H 9.83.

tert-Butyl-3-hydroxynon-8-enoate (**23**)

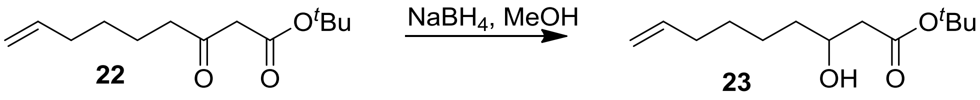

Ester **22** (4.10 g, 18.1 mmol) was dissolved in 50 ml MeOH and the solution cooled to -30 °C. $NaBH_4$ (820 mg, 21.7 mmol) was added in small portions and the reaction mixture stirred for 5 h at the same temperature. After the addition of 50 ml water, the organic phase was separated and the aqueous phase extracted three times with 30 ml EA. The combined organic layers were dried over $MgSO_4$, filtered and the solvent was removed under reduced pressure. After purification by column chromatography on silica (SiO_2, PE:EA, 20:1), compound **23** (3.94 g, 17.3 mmol, 96 %) was obtained as a colorless oil.

R_f (PE:EA, 5:1) = 0.30. IR (Film): $\tilde{\nu}$ = 2978 cm^{-1}, 2932, 2359, 2339, 1729, 1368, 1154; ^1H NMR ($CDCl_3$, 500 MHz): δ = 1.30-1.56 (m, 15 H), 2.03-2.06 (m, 2 H), 2.31 (dd, ^2J = 16.4, ^3J = 9.1 Hz, 1 H), 2.41 (dd, ^2J = 16.4, ^3J = 3.1 Hz, 1 H), 3.11 (bs, 1 H), 3.94-3.98 (m, 1 H), 4.92-5.01 (m, 2 H), 5.75-5.84 (m, 1 H), ^{13}C NMR ($CDCl_3$, 126 MHz): δ = 24.92 (t), 28.10 (q, 3 C), 28.79 (t), 33.65 (t), 36.28 (t), 42.27 (t), 68.03 (d), 81.20 (s), 114.38 (t), 138.81 (d), 172.53 (s); MS (EI(+)): *m/z* (%): 228 (0.1) $[M]^+$, 145 (14), 127 (10), 95 (14), 57 (100), 41 (26); $C_{13}H_{24}O_3$ (228.33): calcd. C 68.38, H 10.59; found C 68.19, H 10.44.

tert-Butyl-3-{[*tert*-butyl(dimethyl)silyl]oxy}non-8-enoate (**24**)

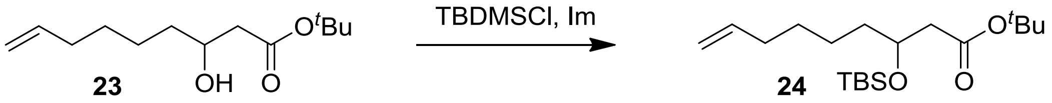

23 **24**

Hydroxyester **23** (3.88 g, 17.0 mmol) was dissolved in 15 ml THF. Imidazole (2.93 g, 43.0 mmol) and TBDMSCl (3.07 g, 20.4 mmol) were added and the solution was stirred for 16 h at rt. After the addition of 50 ml water, the organic phase was separated and the aqueous phase extracted three times with 30 ml EA. The combined organic layers were dried over $MgSO_4$, filtered and the solvent was removed under reduced pressure. After purification by column chromatography on silica (SiO_2, PE:EA, 40:1), compound **24** (5.61 g, 16.4 mmol, 96%) was obtained as a colorless oil.

R_f (PE:EA, 30:1) = 0.26. IR (Film): $\tilde{\nu}$ = 2930 cm^{-1}, 2857, 2415, 2360, 1732, 1465, 1368, 1253, 1156; ^1H NMR ($CDCl_3$, 500 MHz): δ = 0.05 (s, 3 H), 0.06 (s, 3 H), 0.87 (s, 9 H), 1.25-1.52 (m, 15 H), 2.02-2.06 (m, 2 H), 2.32 (dd, ^2J = 14.8, ^3J = 6.1 Hz, 1 H), 2.38 (dd, ^2J = 14.8, ^3J = 6.4 Hz, 1 H), 4.05-4.10 (m, 1 H), 4.92-5.01 (m, 2 H), 5.76-5.84 (m, 1 H); ^{13}C NMR ($CDCl_3$, 126 MHz): δ = -4.64 (q), -4.55 (q), 18.03 (s), 24.51 (t), 25.85 (q, 3 C), 28.15 (q, 3 C), 28.97 (t), 33.72 (t), 37.22 (t), 43.87 (t), 69.29 (d), 80.21 (s), 114.38 (t), 138.90 (d), 171.08 (s); MS (EI(+)): *m/z* (%): 343 (0.1) $[M]^+$, 229 (100), 211 (16), 95 (10), 75 (14), 57 (100), 41 (26); $C_{19}H_{38}O_3Si$ (342.59): calcd. C 66.61, H 11.18; found C 66.70, H 11.23.

3-{[*tert*-Butyl(dimethyl)silyl]oxy}non-8-enal (**25**)

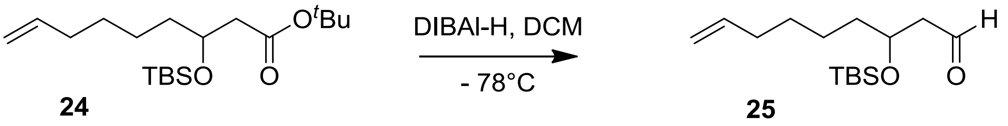

Ester **24** (5.36 g, 15.6 mmol) was dissolved in 30 ml DCM and cooled to –78 °C. DIBAl-H (19 ml, 19.0 mmol, 1 M solution in hexane) was added over 30 min and the reaction stirred for an additional 4.5 h. After the addition of 5 ml MeOH, the cooling bath was removed, 20 ml H_2O were added and the aqueous phase was extracted three times with 50 ml Et_2O. The combined organic layers were dried over $MgSO_4$, filtered and the solvent was removed under reduced pressure. After purification by column chromatography on silica (SiO_2, PE:EA, 50:1), compound **25** (3.51 g, 13.0 mmol, 83%) was obtained as a colorless oil.

R_f (PE:EA, 30:1) = 0.24; IR (Film): $\tilde{\nu}$ = 2929 cm^{-1}, 2856, 2360, 1726, 1463, 1361, 1255, 1102, 910, 836, 776. ^{1}H NMR ($CDCl_3$, 500 MHz): δ = 0.05 (s, 3 H), 0.07 (s, 3 H), 0.87 (s, 9 H), 1.22-1.45 (m, 4 H), 1.46-1.51 (m, 2 H), 2.03-2.07 (m, 2 H), 2.51 (dd, ^3J = 5.8, ^3J = 2.5 Hz, 2 H), 4.18 (m, 1 H), 4.93-5.02 (m, 2 H), 5.75-5.83 (m, 1 H), 9.81 (t, ^3J = 2.5 Hz, 1 H); ^{13}C NMR ($CDCl_3$, 126 MHz): δ = -4.31 (q), -4.04 (q), 17.98 (s), 24.59 (t), 25.76 (q, 3 C), 28.82 (t), 33.64 (t), 37.67 (t), 50.83 (t), 68.22 (d), 114.52 (t), 138.68 (d), 202.31 (d); MS (CI(+)): *m/z* (%): 271 (53) [MH]$^+$, 227 (38), 213 (56), 95 (100), 75 (40); $C_{15}H_{30}O_2Si$ (270.48): calcd. C 66.61, H 11.18; found C 66.58, H 10.70.

3-{[*tert*-Butyl(dimethyl)silyl]oxy}-1-(trimethylsilyl)undec-10-en-1-yn-3-ol (**26**)

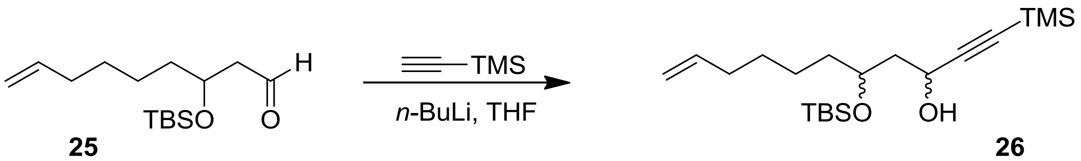

TMS-acetylene (3.70 g, 37.7 mmol) was dissolved in 60 ml THF at –40 °C and *n*-BuLi (37.7 mmol, 23.6 ml, 1.6 M solution in hexane) was added over 15 min. After stirring for 1 h, aldehyde **25** (3.35 g, 12.4 mmol) was added and the solution stirred for an additional hour. After the addition of 30 ml saturated NH_4Cl–solution, the aqueous phase was extracted three times with 50 ml DCM. The combined organic layers were dried over $MgSO_4$, filtered and the solvent was removed under reduced pressure. After purification by column chromatography on silica (SiO_2, PE:EA, 80:1) , compound **26** (two diastereomers, 4.40 g, 11.9 mmol, 96 %) were obtained as yellow oils.

26a: R_f (PE:EA, 10:1) = 0.38. IR (Film): $\tilde{\nu}$ = 2954 cm^{-1}, 2929, 2857, 1250, 1062, 1030, 1005, 835, 774, 760; ^{1}H NMR ($CDCl_3$, 250 MHz): δ = 0.09 (s, 3 H), 0.12 (s, 3 H), 0.17 (s, 9 H), 0.89 (s, 9 H), 1.25-1.58 (m, 6 H), 1.82-1.88 (m, 2 H), 2.01-2.10 (m, 2 H), 3.28 (d, ^3J = 4.0 Hz, 1 H), 4.05-4.14 (m, 1 H), 4.55-4.64 (m, 1 H), 4.91-5.04 (m, 2 H), 5.72-5.88 (m, 1 H); ^{13}C NMR ($CDCl_3$, 63 MHz): δ = -4.57 (q), -4.27 (q), -0.08 (q, 3 C), 17.99 (s), 24.51 (t), 25.89 (q, 3 C), 28.97 (t), 33.71 (t), 36.80 (t), 42.37 (t), 60.47 (d), 70.59 (d), 88.92 (s), 106.80 (s), 114.52 (t), 138.76 (d); MS (FAB(+)): *m/z* (%): 369

(14) [MH]$^+$, 227 (50), 95 (40), 73 (100); $C_{20}H_{40}O_2Si_2$ (368.70): calcd. C 65.15, H 10.94; found C 65.32, H 10.91.

26b: R_f (PE:EA, 10:1) = 0.31; IR (Film): $\tilde{\nu}$ = 2957 cm^{-1}, 2929, 2856, 1250, 1066, 836, 774, 760; ^{1}H NMR ($CDCl_3$, 250 MHz): δ = 0.07 (s, 3 H), 0.09 (s, 3 H), 0.17 (s, 9 H), 0.88 (s, 9 H), 1.25-1.44 (m, 4 H), 1.46-1.58 (m, 2 H), 1.74-1.94 (m, 2 H), 2.01-2.09 (m, 2 H), 2.63 (bs, 1 H), 3.87-3.94 (m, 1 H), 4.52 (t, ^3J = 6.9 Hz, 1 H), 4.91-5.08 (m, 2 H), 5.72-5.88 (m, 1 H); ^{13}C NMR ($CDCl_3$, 63 MHz): δ = -4.56 (q), -4.00 (q), -0.00 (q, 3 C), 18.12 (s), 24.32 (t), 25.99 (q, 3 C), 29.12 (t), 33.87 (t), 37.77 (t), 44.00 (t), 61.91 (d), 71.27 (d), 89.73 (s), 106.66 (s), 114.60 (t), 138.93 (d); MS (CI(+)): *m/z* (%): 369 (47) [MH]$^+$, 227 (18), 187 (17), 95 (100), 75 (40), 75 (14); $C_{20}H_{40}O_2Si_2$ (368.70): calcd. C 65.15, H 10.94; found C 65.31, H 10.89.

N-(3-{[*tert*-Butyl(dimethyl)silyl]oxy}-1-[(trimethylsilyl)ethynyl]non-8-en-1-yl)-4-methyl-*N*-[(5-methylfuran-2-yl)methyl]benzenesulfonamide (**27**)

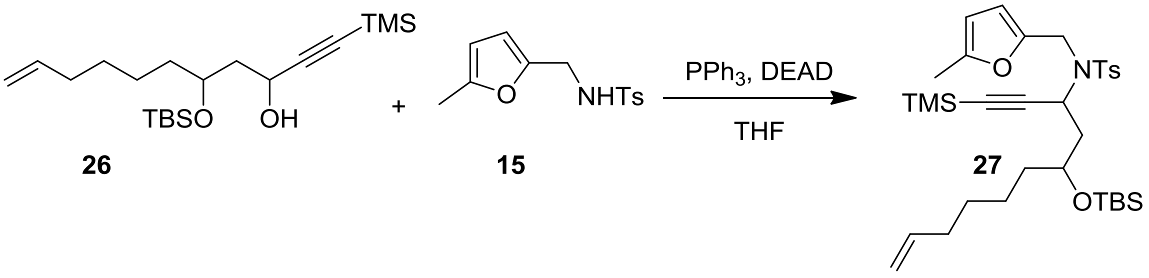

Alcohol **26** (1.53 g, 4.15 mmol), amide **15** (1.43 g, 5.40 mmol), PPh_3 (1.47 g, 5.60 mmol) and DEAD (880 µl, 5.60 mmol) were dissolved in 30 ml THF at 0 °C. The solution was stirred for 16 h at rt. After purification of the crude product by column

chromatography on silica (SiO_2, PE:EA, 100:1), compound **27** (two diastereomers, 2.10 g, 3.41 mmol, 82 %) were obtained as yellow oils.

27a: R_f (PE:EA, 20:1) = 0.19; IR (Film): $\tilde{\nu}$ = 2928 cm^{-1}, 2856, 1598, 1400, 1352, 1250, 1164, 1092, 1060, 838, 775, 760, 666; ^{1}H NMR ($CDCl_3$, 500 MHz): δ = 0.01 (s, 9 H), 0.04 (s, 3 H), 0.07 (s, 3 H), 0.88 (s, 9 H), 1.15-1.49 (m, 6 H), 1.61-1.65 (m, 2 H), 2.01-2.05 (m, 2 H), 2.22 (s, 3 H), 2.41 (s, 3 H), 3.72-3.76 (m, 1 H), 4.12 (d, ^2J = 15.9 Hz, 1 H), 4.50 (d, ^2J = 15.9 Hz, 1 H), 4.72-4.75 (m, 1 H), 4.93-5.01 (m, 2 H), 5.76-5.84 (m, 1 H), 5.86 (d, ^3J = 2.9 Hz, 1 H), 6.14 (d, ^3J = 2.9 Hz, 1 H), 7.28 (d, ^3J = 8.2 Hz, 2 H), 7.72 (d, ^3J = 8.2 Hz, 2 H); ^{13}C NMR ($CDCl_3$, 126 MHz): δ = -4.46 (q), -4.41 (q), -0.32 (q, 3 C), 13.55 (q), 18.10 (s), 21.50 (q), 24.30 (t), 25.96 (q, 3 C), 29.08 (t), 33.75 (t), 36.36 (t), 41.56 (t), 43.00 (t), 48.55 (d), 69.20 (d), 91.10 (s), 101.70 (s), 106.53 (d), 110.26 (d), 114.26 (t), 127.79 (d, 2 C), 129.41 (d, 2 C), 136.26 (s), 138.98 (d), 143.18 (s), 148.82 (s), 151.72 (s); MS (FAB(+)): *m/z* (%): 614 (4) [M-H]$^+$, 558 (5), 460 (14), 294 (31), 227 (14), 95 (100), 73 (88); $C_{33}H_{53}NO_4SSi_2$ (616.02): calcd. C 64.34, H 8.67, N 2.27; found C 64.52, H 8.77, N 2.16.

27b: R_f (PE:EA, 20:1) = 0.17. IR (Film): $\tilde{\nu}$ = 2930 cm^{-1}, 2856, 1352, 1330, 1250, 1164, 838, 775, 659. ^{1}H NMR ($CDCl_3$, 500 MHz): δ = 0.01 (s, 9 H), 0.04 (s, 3 H), 0.08 (s, 3 H), 0.92 (s, 9 H), 1.08-1.65 (m, 8 H), 1.99-2.04 (m, 2 H), 2.22 (s, 3 H), 2.41 (s, 3 H), 3.70-3.75 (m, 1 H), 4.16 (d, ^2J = 15.9 Hz, 1 H), 4.55 (d, ^2J = 15.9 Hz, 1 H), 4.89-5.01 (m, 3 H), 5.74-5.83 (m, 1 H), 5.86 (d, ^3J = 3.0 Hz, 1 H), 6.12 (d, ^3J = 3.0 Hz, 1 H), 7.26 (d, ^3J = 8.2 Hz, 2 H), 7.69 (d, ^3J = 8.2 Hz, 2 H);^{13}C NMR ($CDCl_3$, 126 MHz): δ = -4.81 (q), -4.16 (q), -0.31 (q, 3 C), 13.49 (q), 18.04 (s), 21.48 (q), 23.92 (t), 25.92 (q, 3 C), 29.93 (t), 33.71 (t), 37.43 (t), 41.86 (t, 2 C), 49.15 (d), 69.96 (d), 91.46 (s), 101.57

(s), 106.40 (d), 110.20 (d), 114.37 (t), 127.73 (d, 2 C), 129.32 (d, 2 C), 136.57 (s), 138.80 (d), 143.00 (s), 148.94 (s), 151.58 (s); MS (FAB(+)): *m/z* (%): 614 (3), 558 (8), 460 (12), 294 (20), 227 (10), 95 (100), 73 (57); $C_{33}H_{53}NO_4SSi_2$ (616.02): calcd. C 64.34, H 8.67, N 2.27; found C 64.74, H 8.78, N 2.15.

N-(3-{[*tert*-Butyl(dimethyl)silyl]oxy}-1-[(trimethylsilyl)ethynyl]non-8-en-1-yl)-4-methyl-*N*-[(5-methylfuran-2-yl)methyl]benzenesulfonamide (**31**)

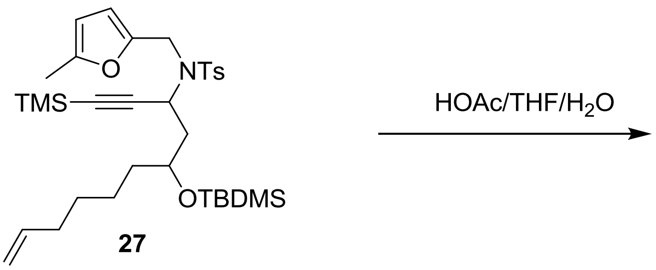

Compound **27** (900 mg, 1.46 mmol) was dissolved in 20 ml THF/H_2O (1:1) at 0 °C and HOAc (30 ml) added. After stirring for 72 h at rt, 20 ml DCM was added and the solution extracted twice with 20 ml DCM. The combined organic layers were dried over $MgSO_4$, filtered and the solvent was removed under reduced pressure. After purification by column chromatography on silica (SiO_2, PE:EA, 20:1), compound **31** (two diastereomers, 735 mg, 1.46 mmol, 100 %) were obtained as a colorless solids.

31a: Mp: 64 °C; R_f (PE:EA, 10:1) = 0.24; IR (Film): $\tilde{\nu}$ = 3527 cm^{-1}, 2931, 1358, 1331, 1160, 1095, 1060, 843, 810; ^{1}H NMR ($CDCl_3$, 500 MHz): δ = -0.01 (s, 9 H), 1.12-1.27 (m, 2 H), 1.31-1.44 (m, 6 H), 2.01-2.04 (m, 2 H), 2.25 (s, 3 H), 2.43 (s, 3 H), 3.66-3.70 (m, 1 H), 4.11 (d, ^2J = 15.3 Hz, 1 H), 4.61 (d, ^2J = 15.3 Hz, 1 H), 4.86-5.01 (m, 3 H), 5.76-5.84 (m, 1 H), 5.89 (d, ^3J = 3.0 Hz, 1 H), 6.16 (d, ^3J = 3.0 Hz, 1 H),

7.32 (d, ^3J = 8.2 Hz, 2 H), 7.72 (d, ^3J = 8.2 Hz, 2 H);* ^{13}C NMR ($CDCl_3$, 126 MHz): δ = -0.37 (q, 3 C), 13.57 (q), 21.50 (q), 25.36 (t), 29.11 (t), 33.75 (t), 36.72 (t), 41.57 (t), 42.42 (t), 48.35 (d), 66.52 (d), 90.98 (s), 101.01(s), 106.58 (d), 110.80 (d), 114.26 (t), 127.43 (d, 2 C), 129.67 (d, 2 C), 135.52 (s), 138.98 (d), 143.62 (s), 148.34 (s), 152.04 (s); MS (EI(+)): *m/z* (%): 501 (1) $[M]^+$, 346 (85), 264 (25), 110 (21), 95 (100), 73 (12). $C_{27}H_{39}NO_4SSi$ (501.75): calcd. C 64.63, H 7.83, N 2.79; found C 64.44, H 7.86, N 2.74. *OH-groups not detected due to H/D exchange

31b: Mp.: 69 °C; R_f (PE:EA, 10:1) = 0.08; IR (Film): $\tilde{\nu}$ = 3507cm^{-1}, 2930, 1331, 1250, 1163, 1089, 1017, 840, 788. ^{1}H NMR ($CDCl_3$, 500 MHz): δ = 0.01 (s, 9 H), 1.28-1.43 (m, 6 H), 1.55-1.64 (m, 2 H),1.68 (bs, 1 H), 2.02-2.06 (m, 2 H), 2.24 (s, 3 H), 2.42 (s, 3 H), 3.65-3.70 (m, 1 H), 4.16 (d, ^2J = 15.7 Hz, 1 H), 4.54 (d, ^2J = 15.7 Hz, 1 H), 4.92 -5.01 (m, 3 H), 5.75-5.82 (m, 1 H), 5.87 (d, ^3J = 3.0 Hz, 1 H), 6.15 (d, ^3J = 3.0 Hz, 1 H), 7.28 (d, ^3J = 8.2 Hz, 2 H), 7.73 (d, ^3J = 8.2 Hz, 2 H);^{13}C NMR ($CDCl_3$, 126 MHz): δ = -0.33 (q, 3 C), 13.51 (q), 21.48 (q), 24.85 (t), 28.75 (t), 33.66 (t), 37.19 (t), 41.74 (t), 42.85 (t), 49.15 (d), 68.95 (d), 91.58 (s), 101.09(s), 106.46 (d), 110.45 (d), 114.38 (t), 127.71 (d, 2 C), 129.45 (d, 2 C), 136.16 (s), 138.82 (d), 143.28 (s), 148.61 (s), 151.87 (s); MS (FAB(+)): *m/z* (%): 502 (9) $[MH]^+$, 342 (45), 95 (100), 73 (6); $C_{27}H_{39}NO_4SSi$ (501.75): calcd. C 64.63, H 7.83, N 2.79; found C 64.45, H 7.81, N 2.75.

4-Methyl-*N*-[(5-methylfuran-2-yl)methyl]-*N*-{3-oxo-1-[(trimethylsilyl)ethynyl]non-8-en-1-yl}benzenesulfonamide (**32**)

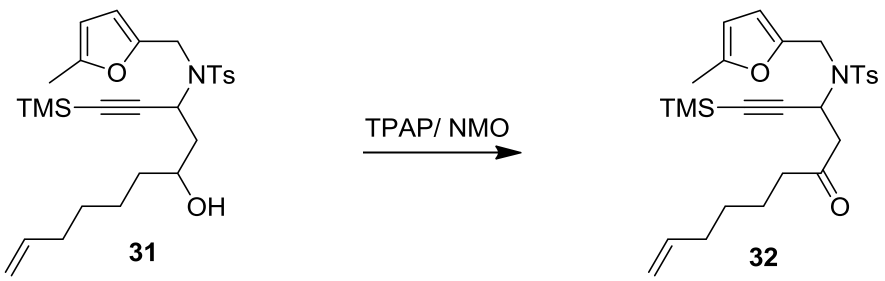

Alcohol **31** (50.0 mg, 100 mmol) was dissolved in 5 ml DCM and molecular sieve (4 Å) added. At 0 °C, TPAP (1.80 mg, 5.10 µmol) and NMO (58.6 mg, 500 µmol) were added and the solution was stirred for 16 h at rt. After filtration through celite, the solution was dried over $MgSO_4$, filtered and evaporated. After purification by column chromatography on silica (SiO_2, PE:EA, 10:1), compound **32** (40.1 mg, 80 µmol, 80 %) was isolated as a colorless solid.

Mp.: 34 °C; R_f (PE:EA, 5:1) = 0.21 ; IR (Film): $\tilde{\nu}$ = 2938 cm^{-1}, 1721, 1354, 1327, 1165, 1092, 905, 876, 838, 665 ; ^1H NMR ($CDCl_3$, 500 MHz): δ = 0.00 (s, 9 H), 1.31-1.37 (m, 2 H), 1.49-1.55 (m, 2 H), 2.01-2.05 (m, 2 H), 2.26 (s, 3 H), 2.27-2.29 (m, 2 H), 2.42 (s, 3 H), 2.51 (dd, ^2J = 16.5 Hz, ^3J = 8.4 Hz, 1 H), 2.63 (dd, ^2J = 16.5 Hz, ^3J = 5.8 Hz, 1 H), 4.13 (d, ^2J = 15.6 Hz, 1 H), 4.57 (d, ^2J = 15.6 Hz, 1 H), 4.93-5.02 (m, 2 H), 5.24 (dd, ^3J = 8.4 Hz, ^3J = 5.8 Hz, 1 H), 5.73-5.81 (m, 1 H), 5.90 (d, ^3J = 3.1 Hz, 1 H), 6.14 (d, ^3J = 3.1 Hz, 1 H), 7.28 (d, ^3J = 8.2 Hz, 2 H), 7.74 (d, ^3J = 8.2 Hz, 2 H) ; ^{13}C NMR ($CDCl_3$, 126 MHz): δ = 0.00 (q, 3 C), 13.94 (q), 21.88 (q), 23.27 (t), 28.69 (t), 33.84 (t), 42.42 (t), 43.35 (t), 47.38 (d), 48.46 (t), 91.73 (s), 100.90 (s), 106.95 (d), 111.05 (d), 115.04 (t), 128.17 (d, 2 C), 129.86 (d, 2 C), 136.43 (s), 138.79 (d), 143.76

(s), 148.81 (s), 152.43 (s), 206.33 (s); MS (EI(+)): *m/z* (%): 499 (0.3) $[M]^+$, 344 (100), 218 (10), 110 (11), 95 (30); $C_{27}H_{37}NO_4SSi$ (499.74): calcd. C 64.89, H 7.46, N 2.80; found C 64.79, H 7.42, N 2.73.

N-(1-Ethynyl-3-hydroxynon-8-en-1-yl)-4-methyl-*N*-[(5-methylfuran-2-yl)methyl]benzenesulfonamide (**28**)

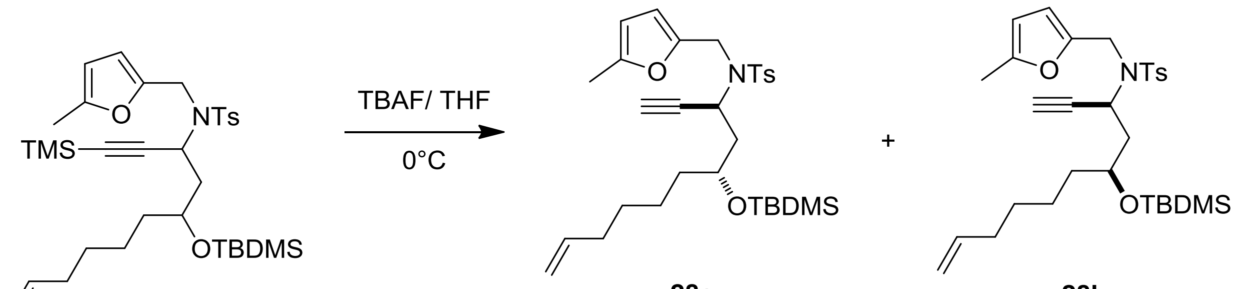

27 **28a** **28b**

Amide **27** (616 mg, 1.00 mmol) was dissolved in 20 ml DCM and TBAF (789 mg, 2.50 mmol) added at 0 °C. After 30 min, 30 ml saturated NH_4Cl-solution and 20 ml EA were added and the aqueous phase was extracted three times with 20 ml EA. The combined organic layers were dried over $MgSO_4$, filtered and the solvent was removed under reduced pressure. After purification by column chromatography on silica (SiO_2, PE:EA, 10:1), **28** (540 mg, 990 µmol, 99%) was obtained as mixture of diasteromers. For pure diasteromers, additional experiments were run with diasteromerically pure starting materials. The relative configuration of **28b** was assigned via X-ray crystal structure analysis.

28a: colorless oil; R_f (PE:EA, 3:1) = 0.56; IR (Film): $\tilde{\nu}$ = 3276 cm^{-1}, 2928, 2856, 1351, 1160, 1048, 940, 833, 775, 662, 581; ^{1}H NMR ($CDCl_3$, 300 MHz): δ = 0.04 (s, 3 H), 0.08 (s, 3 H), 0.88 (s, 9 H), 1.17-1.45 (m, 6 H), 1.67-1.71 (m, 2 H), 2.00-2.06 (m, 2 H), 2.15 (d, ^4J = 2.3 Hz, 1 H), 2.21 (s, 3 H), 2.42 (s, 3 H), 3.71-3.77 (m, 1 H), 4.17 (d, ^2J = 16.0 Hz, 1 H), 4.51 (d, ^2J = 16.0 Hz, 1 H), 4.78 (td, ^3J = 7.3 Hz, ^4J = 2.3 Hz, 1 H), 4.93-5.01 (m, 2 H), 5.73-5.84 (m, 1 H), 5.85-5.86 (m, 1 H), 6.14 (d, ^3J = 3.0 Hz, 1 H), 7.28 (d, ^3J = 8.2 Hz, 2 H), 7.71 (d, ^3J = 8.2 Hz, 2 H); ^{13}C NMR ($CDCl_3$, 126 MHz): δ = -4.47 (q), -4.36/-4.19 (q), 13.51 (q), 18.07 (s), 21.52 (q), 24.08 (t), 25.93 (q, 3 C), 29.08 (t), 33.70 (t), 36.37 (t), 41.45 (t), 42.74 (t), 47.65 (d), 69.02 (d), 74.10 (d), 80.27 (s),106.50 (d), 110.29 (d), 114.28 (t), 127.72 (d, 2 C), 129.38 (d, 2 C), 136.30 (s), 138.94 (d), 143.32 (s), 148.65 (s), 151.72 (s); MS (EI(+)): *m/z* (%): 543 [M]$^+$, 392 (34), 330 (10), 293 (10), 227 (13), 220 (23), 205 (49), 95 (100), 73 (21), 57 (14); $C_{30}H_{45}NO_4SSi$ (543.83): calcd. C 66.26, H 8.34, N 2.58; found C 66.37, H 8.36, N 2.53.

28b: colorless solid; Mp.: 48 °C; R_f (PE:EA, 3:1) = 0.56; IR (Film): $\tilde{\nu}$ = 3275 cm^{-1}, 2925, 2855, 1352, 1329, 1249, 1162, 1094, 938, 832, 773, 663; ^{1}H NMR ($CDCl_3$, 500 MHz): δ = 0.04 (s, 3 H), 0.08 (s, 3 H), 0.91 (s, 9 H), 1.11-1.26 (m, 2 H), 1.29-1.35 (m, 2 H), 1.37-1.46 (m, 2 H), 1.56-1.59 (m, 2 H), 1.99-2.03 (m, 2 H), 2.17 (d, ^4J = 2.3 Hz, 1 H), 2.19 (s,3 H), 2.41 (s, 3 H), 3.71-3.77 (m, 1 H), 4.23 (d, ^2J = 16.0 Hz, 1 H), 4.58 (d, ^2J = 16.0 Hz, 1 H), 4.90-4.92 (m, 1 H), 4.93-5.01 (m, 2 H), 5.74-5.82 (m, 1 H), 5.85 (d, ^3J = 3.0 Hz, 1 H), 6.13 (d, ^3J = 3.0 Hz, 1 H), 7.26 (d, ^3J = 8.2 Hz, 2 H), 7.69 (d, ^3J = 8.2 Hz, 2 H);^{13}C NMR ($CDCl_3$, 126 MHz): δ = -4.79 (q), -4.19 (q), 13.45 (q), 18.00 (s), 21.52 (q), 23.87 (t), 25.88 (q, 3 C), 29.02 (t), 33.71 (t), 36.47 (t), 41.70 (t), 42.79 (t), 48.13 (d), 68.86 (d), 74.43 (d), 80.18 (s),106.37 (d), 110.31 (d), 114.37 (t), 127.64 (d,

2 C), 129.29 (d, 2 C), 136.81 (s), 138.80 (d), 143.32 (s), 148.74 (s), 151.62 (s); MS (CI(+)): *m/z* (%): 544 (7) $[MH]^+$, 486 (93), 462 (21), 392 (92), 388 (63), 303 (10), 221 (23), 95 (100); $C_{30}H_{45}NO_4SSi$ (543.83): calcd. C 66.26, H 8.34, N 2.58; found C 66.37, H 8.36, N 2.53.

N-(1-Ethynyl-3-hydroxynon-8-en-1-yl)-4-methyl-*N*-[(5-methylfuran-2-yl)methyl]benzenesulfonamide (**29**)

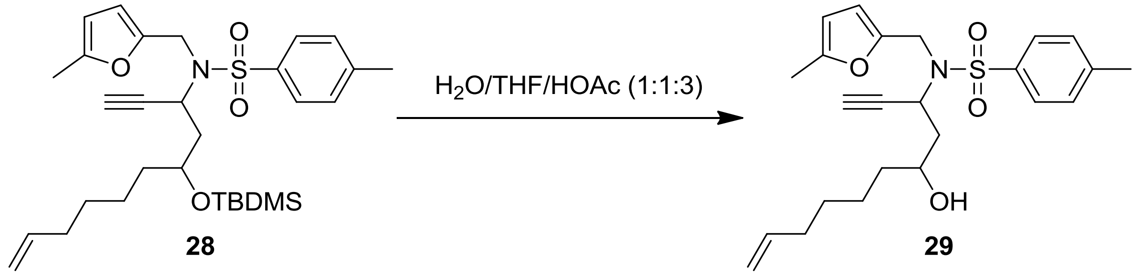

Compound **28** (534 mg, 980 µmol) was dissolved in 6 ml H_2O/ THF (1:1) and the solution cooled to 0°C. Acetic acid (9 ml) was added and the solution stirred for 5 d at rt. After the addition of 20 ml EA, the layers were separated and the aqueous phase was extracted three times with 20 ml EA. The combined organic layers were dried over $MgSO_4$, filtered and the solvent was removed under reduced pressure. After purification by column chromatography on silica (SiO_2, PE:EA, 20:1), **29** (two diastereomers, 386 mg, 900 µmol, 92%) were obtained as colorless solid and as a colorless oil.

29a: Mp.: 56 °C; R_f (PE:EA, 10:1) = 0.14. IR (Film): $\tilde{\nu}$ = 3538 cm^{-1}, 3259, 2928, 2362, 1330, 1157, 1101, 1089, 1000, 972, 937, 908, 892, 870, 816, 704, 578. ^1H

NMR ($CDCl_3$, 500 MHz): δ = 1.12-1.27 (m, 2 H), 1.31-1.43 (m, 4 H), 1.45-1.56 (m, 2 H), 2.01-2.05 (m, 2 H), 2.14 (d, ^4J = 2.4 Hz, 1 H), 2.25 (s, 3 H), 2.44 (s, 3 H), 2.90 (bs, 1 H), 3.62-3.69 (m, 1 H), 4.17 (d, ^2J = 15.6 Hz, 1 H), 4.62 (d, ^2J = 15.6 Hz, 1H), 4.90-5.02 (m, 3 H), 5.76-5.84 (m, 1 H), 5.89 (d, ^3J = 3.0 Hz, 1 H), 6.19 (d, ^3J = 3.0 Hz, 1 H), 7.32 (d, ^3J = 8.2 Hz, 2 H), 7.73 (d, ^3J = 8.2 Hz, 2 H);^{13}C NMR ($CDCl_3$, 126 MHz): δ = 13.55 (q), 21.54 (q), 24.32 (t), 29.07 (t), 33.73 (t), 36.36 (t), 41.49 (t), 42.64 (t), 47.49 (d), 66.41 (d), 74.13 (d), 79.64 (s), 106.62 (d), 110.80 (d), 114.28 (t), 127.35 (d, 2 C), 129.70 (d, 2 C), 135.78 (s), 138.95 (d), 143.73 (s), 148.33 (s), 152.02 (s). MS (EI(+)): *m/z* (%): 429 (5) $[M]^+$, 274 (100), 192 (12), 148 (12), 95 (93), 91 (15); $C_{24}H_{31}NO_4S$ (429.75): calcd. C 67.10, H 7.27, N 3.26; found C 67.05 H 7.29, N 3.18.

29b: R_f (PE:EA, 10:1) = 0.07; IR (Film): $\tilde{\nu}$ = 3819 cm^{-1}, 2926, 2360, 1698, 1558, 1540, 1521, 1507,1161, 897, 632. ^{1}H NMR ($CDCl_3$, 500 MHz): δ = 1.34-1.42 (m, 6 H), 1.59-1.69 (m, 2 H), 2.02-2.06 (m, 2 H), 2.20 (d, ^4J = 2.5 Hz, 1 H), 2.22 (s, 3 H), 2.42 (s, 3 H), 3.66-3.71 (m, 1 H), 4.23 (d, ^2J = 15.9 Hz, 1 H), 4.55 (d, ^2J = 15.9 Hz, 1H), 4.92-5.03 (m, 3 H), 5.75-5.83 (m, 1 H), 5.87 (d, ^3J = 3.0 Hz, 1 H), 6.16 (d, ^3J = 3.0 Hz, 1 H), 7.28 (d, ^3J = 8.2 Hz, 2 H), 7.72 (d, ^3J = 8.2 Hz, 2 H);* ^{13}C NMR ($CDCl_3$, 126 MHz): δ = 13.47 (q), 21.51 (q), 24.51 (t), 28.78 (t), 33.65 (t), 37.38 (t), 41.65 (t), 42.77 (t), 48.13 (d), 68.81 (d), 74.56 (d), 79.74 (s), 106.45 (d), 110.51 (d), 114.42 (t), 127.63 (d, 2 C), 129.43 (d, 2 C), 136.49 (s), 138.78 (d), 143.37 (s), 148.50 (s), 151.87 (s); MS (CI(+)): *m/z* (%): 430 (13) $[MH]^+$, 348 (10), 274 (83), 148 (12), 95 (100), 91 (9); HRMS (CI (+)): $C_{24}H_{31}NO_4S$: calcd. 429.1974; found 429.1971.*OH-groups not detected due to H/D exchange

N-(1-Ethynyl-3-oxonon-8-en-1-yl)-4-methyl-*N*-[(5-methylfuran-2-yl)methyl]benzenesulfonamide (**16**)

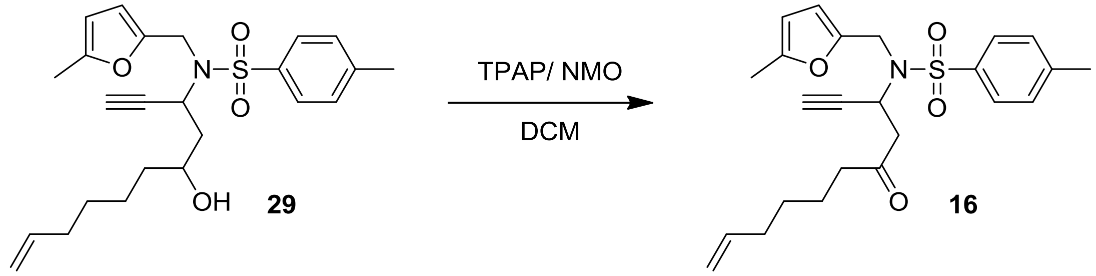

Alcohol **29** (150 mg, 350 µmol) was dissolved in 10 ml DCM at 0 °C and molecular sieve added. NMO (205 mg, 1.75 mmol) and TPAP (6.30 mg, 1.80 µmol) were added and after 16 h at rt the solution was filtered through celite. The filtrate was dried over $MgSO_4$, filtered and the solvent was removed under reduced pressure. After purification by column chromatography on silica (SiO_2, PE:EA, 15:1), **16** (113 mg, 260 µmol, 76%) was obtained as a yellow solid.

Mp.: 44 °C; R_f (PE:EA, 10:1) = 0.11; IR (fest): $\tilde{\nu}$ = 3269 cm^{-1}, 2925, 1716, 1355, 1162, 1093, 812; ^{1}H NMR (acetone-d$_6$, 500 MHz): δ = 1.29-1.36 (m, 2 H), 1.46-1.52 (m, 2 H), 2.00-2.05 (m, 2 H), 2.22 (s, 3 H), 2.23-2.36 (m, 2 H), 2.43 (s, 3 H), 2.66 (dd, ^2J = 16.9 Hz, ^3J = 5.9 Hz, 1 H), 2.71 (dd, ^2J = 16.9 Hz, ^3J = 8.2 Hz, 1 H), 2.77 (d, ^4J = 2.4 Hz, 1 H), 4.24 (d, ^2J = 15.9 Hz, 1 H), 4.60 (d, ^2J = 15.9 Hz, 1 H), 4.91-5.01 (m, 2 H), 5.28 (ddd, ^3J = 8.2 Hz, ^3J = 5.9 Hz, ^4J = 2.4 Hz, 1 H), 5.75-5.83 (m, 1 H), 5.97 (d, ^3J = 3.0 Hz, 1 H), 6.19 (d, ^3J = 3.0 Hz, 1 H), 7.40 (d, ^3J = 8.2 Hz, 2 H), 7.74 (d, ^3J = 8.2 Hz, 2 H);^{13}C NMR (acetone-d$_6$, 126 MHz): δ = 13.38 (q), 21.32 (q), 23.44 (t), 28.89 (t), 34.08 (t), 42.40 (t), 43.05 (t), 46.54 (d), 48.34 (t), 75.83 (d), 80.22 (s), 107.25 (d), 111.40 (d), 114.83 (t), 128.41 (d, 2 C), 130.26 (d, 2 C), 137.57 (s), 139.35 (d), 144.37

(s), 149.34 (s), 152.70 (s), 206.16 (s); MS (EI(+)): *m/z* (%): 427 $[M]^+$, 272 (100), 160 (14), 110 (10), 95 (38), 91 (10); HRMS (EI (+)): $C_{24}H_{29}NO_4S$: calcd. 427.1817; found 427.1796.

1-{7-Hydroxy-6-methyl-2-[(4-methylphenyl)sulfonyl]-2,3-dihydro-1*H*-isoindol-1-yl}oct-7-en-2-on (**18**)

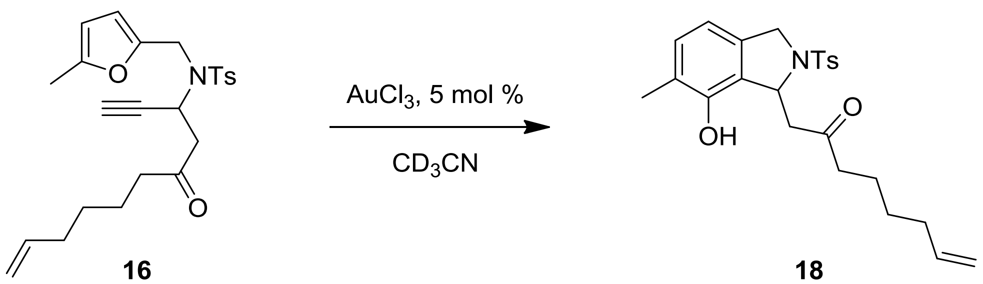

Compound **16** (40.0 mg, 93.6 µmol) and $AuCl_3$ (1.42 mg, 4.68 µmol) were dissolved in 1 ml CD_3CN and allowed to react for 30 min at rt. After evaporation of the solvent, the crude product was purified by column chromatography on silica (SiO_2, PE:EA, 10:1) to afford **18** (37.4 mg, 87.5 µmol, 93%) as a colorless oil.

R_f (PE:EA, 10:1) = 0.13; IR (Film): $\tilde{\nu}$ = 3260 cm^{-1}, 2924, 1711, 1640, 1598, 1345, 1307, 1161, 1094, 1051, 664; ^1H NMR ($CDCl_3$, 300 MHz): δ = 1.37-1.47 (m, 2 H), 1.62-1.72 (m, 2 H), 2.08 (m, 2 H), 2.15 (s, 3 H), 2.36 (s, 3 H), 2.50-2.72 (m, 2 H), 3.03 (dd, ^2J = 18.8 Hz, ^3J = 8.6 Hz, 1 H), 3.74 (dd, ^2J = 18.8 Hz, ^3J = 1.5 Hz, 1 H), 4.44 (d, ^2J = 14.1 Hz, 1 H), 4.69 (d, ^2J = 14.1 Hz, 1 H), 4.95-5.06 (m, 2 H), 5.21 (d, ^3J = 8.6 Hz, 1 H), 5.73-5.87 (m, 1 H), 6.52 (d, ^3J = 7.5 Hz, 1 H), 6.96 (d, ^3J = 7.5 Hz, 1 H), 7.24 (d, ^3J = 8.3 Hz, 2 H), 7.64 (d, ^3J = 8.3 Hz, 2 H), 8.27 (s, 1 H);^{13}C NMR ($CDCl_3$, 126 MHz): δ = 15.74 (q), 21.51 (q), 22.94 (t), 28.24 (t), 33.43 (t), 43.31 (t), 52.46 (t), 53.25

(t), 59.57 (d), 113.09 (d), 114.89 (t), 124.71 (s), 124.95 (s), 127.28 (d, 2 C), 129.95 (d, 2 C), 131.26 (d), 134.03 (s), 134.89 (s), 138.28 (d), 143.93 (s), 149.80 (s), 216.12 (s); MS (EI(+)): *m/z* (%): 427 (2) $[M]^+$, 302 (32), 272 (100), 162 (15), 91 (52). $C_{24}H_{29}NO_4S$ (427.56): calcd. C 67.42, H 6.84, N 3.28; found C 67.25 H 7.21, N 3.03.

3-(2-{[*tert*-Butyl(dimethyl)silyl]oxy}oct-7-en-1-yl)-5-methyl-2-[(4-methylphenyl)sulfonyl]-2,3-dihydro-1-isoindol-4-ol (**37a/37b**) and 3-(2-hydroxyoct-7-en-1-yl)-5-methyl-2-[(4-methylphenyl)sulfonyl]-2,3-dihydro-1-isoindol-4-ol (**38**)

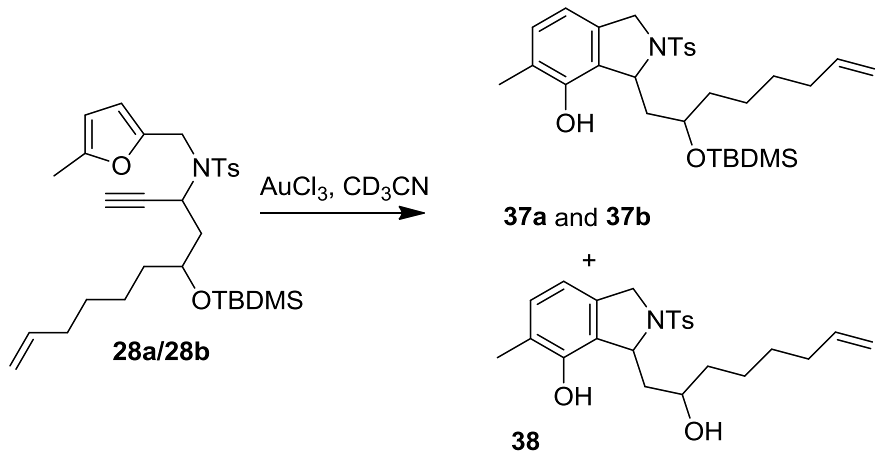

For these transformations the diastereomerically pure compounds were used.

Conversion of **28a**: Compound **28a** (60 mg, 110 µmol) and $AuCl_3$ (1.67 mg, 5.50 µmol) in 2 ml CD_3CN were allowed to react for 30 min at rt. After evaporation of the solvent, the crude product was purified by column chromatography on silica (SiO_2, PE:EA, 5:1) to give diasteromerically pure **37a** (34.8 mg, 64.0 µmol, 58%)

along with a small amount of the deprotected phenol **38** (3.7 mg, 8.6 µmol, 8%) as colorless oils**.**

37a: R_f (PE:EA, 10:1) = 0.28; IR (Film): $\tilde{\nu}$ = 3231 cm^{-1}, 2929, 2858, 2560, 2340, 1599, 1464, 1347, 1310, 1255, 1220, 1162, 1095, 1031, 933, 836, 809, 783, 664, 589; ^{1}H NMR ($CDCl_3$, 500 MHz): δ = 0.22 (s, 3 H), 0.28 (s, 3 H), 0.97 (s, 9H), 1.31-1.46 (m, 4 H), 1.49-1.56 (m, 1 H), 1.66-1.73 (m, 1 H), 2.03-2.08 (m, 2 H), 2.09-2.14 (m, 1 H), 2.16 (s, 3 H), 2.39 (s, 3 H), 2.59 (dt, ^2J = 15.4 Hz, ^4J = 2.7 Hz, 1 H), 4.44 (d, ^2J = 13.3 Hz, 1 H), 4.44-4.49 (m, 1 H), 4.69 (dd, ^2J = 13.3 Hz, ^4J = 2.1 Hz, 1 H), 4.89 (m, 1 H), 4.93-5.03 (m, 2 H), 5.76-5.84 (m, 1 H), 6.51 (d, ^3J = 7.4 Hz, 1 H), 6.98 (d, ^3J = 7.4 Hz, 1 H), 7.26 (d, ^3J = 8.2 Hz, 2 H), 7.71 (d, ^3J = 8.2 Hz, 2 H), 8.48 (bs, 1 H); ^{13}C NMR ($CDCl_3$, 126 MHz): δ = -4.07 (q), -3.81 (q), 15.74 (q), 18.47 (s), 21.63 (q), 23.94 (t), 26.17 (q, 3 C), 29.11 (t), 33.73 (t), 37.69 (t), 45.08 (t), 54.22 (t), 62.89 (d), 74.58 (d), 112.52 (d), 114.68 (t), 124.76 (s), 125.90 (s), 127.69 (d, 2 C), 129.94 (d, 2 C), 131.03 (d), 133.75 (s), 134.34 (s), 138.86 (d), 143.88 (s), 149.86 (s).MS (CI(+)): *m/z* (%): 544 $[MH]^+$ (13), 486 (100), 412 (23), 388 (39), 302 (77), 256 (6). HRMS (CI (+)): $C_{30}H_{45}NO_3SSi$: calcd. 543.2839; found 543.2850.

38: R_f (PE:EA, 5:1) = 0.15; IR (Film): $\tilde{\nu}$ = 2928 cm^{-1}, 2857, 2361, 2341, 1598, 1462, 1434, 1342, 1159, 1094, 664, 634. ^{1}H NMR ($CDCl_3$, 500 MHz): δ = 1.39-1.48 (m, 4 H), 1.54-1.58 (m, 2 H), 2.05-2.11 (m, 3 H), 2.16 (s, 3 H), 2.31-2.33 (m, 1 H), 2.35 (s, 3 H), 2.99 (bs, 1 H), 4.35-4.39 (m, 1 H), 4.45 (d, ^2J = 13.9 Hz, 1 H), 4.68 (d, ^2J = 13.9 Hz, 1 H), 4.94-5.03 (m, 2 H), 5.08-5.10 (m, 1 H), 5.76-5.84 (m, 1 H), 6.52 (d, ^3J = 7.5 Hz, 1 H), 6.96 (d, ^3J = 7.4 Hz, 1 H), 7.23 (d, ^3J = 8.2 Hz, 2 H), 7.69 (d, ^3J = 8.2 Hz, 2 H), 8.29 (bs, 1 H); ^{13}C NMR ($CDCl_3$, 126 MHz): δ = 15.81 (q), 21.63 (q), 24.93 (t), 28.97 (t), 33.76 (t), 37.51 (t), 45.64 (t), 53.88 (t), 62.57 (d), 72.61 (d), 113.06 (d),

114.75 (t), 124.43 (s), 126.39 (s), 127.54 (d, 2 C), 129.99 (d, 2 C), 134.42 (s), 134.44 (s), 138.85 (d), 143.86 (s), 149.51 (s)*; MS (EI(+)): *m/z* (%): 429 $[M]^+$, 302 (100), 274 (29), 160 (10), 155 (18), 148 (33), 91 (41). HRMS (EI (+)): $C_{24}H_{31}NO_4S$: calcd. 429.1974; found 429.1975.* one carbon not detectable, due to the low concentration of the sample

Conversion of **28b**: Compound**28b** (12 mg, 22.0 µmol) and $AuCl_3$ (330 µg, 1.10 µmol) in 500 µl CD_3CN were allowed to react for 30 min at room temperature. After evaporation of the solvent, the crude product was purified by column chromatography on silica (SiO_2, PE:EA, 5:1) to afford diasteromerically pure **37b** (5.60 mg, 10.3 µmol, 47%) as a colorless oil.

R_f (PE:EA, 10:1) = 0.30; IR (Film): $\tilde{\nu}$ = 2930 cm^{-1}, 2857, 2359, 2340, 1597, 1489, 1471, 1346, 1305, 1254, 1160, 1093, 837, 665; ^1H NMR ($CDCl_3$, 500 MHz): δ = 0.09 (s, 3 H), 0.26 (s, 3 H), 0.91 (s, 9 H), 1.35-1.46 (m, 4 H), 1.74-1.85 (m, 1 H), 1.99 (ddd, ^2J = 14.1 Hz, ^3J = 10.1 Hz, ^3J = 3.7 Hz, 1 H), 2.05-2.09 (m, 1 H), 2.15 (s, 3 H), 2.32 (s, 3 H), 2.47 (ddd, ^2J = 14.1 Hz, ^3J = 11.7 Hz, ^3J = 3.7 Hz, 1 H), 3.96 (m, 1 H), 4.52 (d, ^2J = 14.9 Hz, 1 H), 4.66 (d, ^2J = 14.9 Hz, 1 H), 4.94-5.03 (m, 2 H), 5.19 (ddd, ^3J = 10.1 Hz, ^3J = 3.7 Hz, J = 1.7 Hz, 1 H), 5.76-5.84 (m, 1 H), 6.54 (d, ^3J = 7.4 Hz, 1 H), 6.92 (d, ^3J = 7.4 Hz, 1 H), 7.15 (d, ^3J = 8.2 Hz, 2 H), 7.59 (d, ^3J = 8.2 Hz, 2 H), 8.00 (bs, 1 H); ^{13}C NMR ($CDCl_3$, 126 MHz): δ = -3.43 (q), -3.25 (q), 16.01 (q), 18.53 (s), 21.59 (q), 25.87 (t), 26.06 (q, 3 C), 29.09 (t), 33.79 (t), 36.28 (t), 44.76 (t), 52.99 (t), 61.23 (d), 72.95 (d), 113.93 (d), 114.73 (t), 125.34 (s), 127.08 (s), 127.14 (d, 2 C), 129.77 (d, 2 C), 130.87 (d), 135.28 (s), 136.00 (s), 138.86 (d), 143.49 (s), 150.16 (s); MS

(CI(+)): *m/z* (%): 544 $[MH]^+$ (14), 486 (100), 412 (20), 388 (18), 302 (73); HRMS (CI (+)): $C_{30}H_{45}NO_3SSi$: calcd. 543.2839; found 543.2847.

3-[(4-Methoxybenzyl)oxy]-1-(5-methylfuran-2-yl)propan-1-ol (**44**)

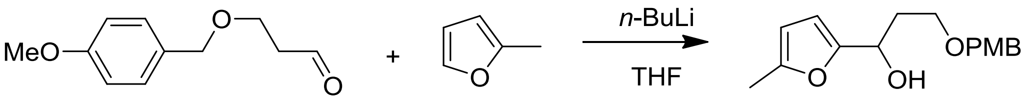

43 **42** **44**

2-Methylfuran (**42**) (2.00 g, 24.4 mmol) was dissolved in 30 ml THF and cooled to -78 °C. *n*-BuLi (16.7 ml, 26.8 mmol, 1.6 M solution in hexane) was added and the cooling bath removed. The reaction was stirred for 4h at rt, re-cooled to -78 °C and 3-(4-methoxybenzyloxy)propanal (**43**) (3.50 g, 18.0 mmol) in 5 ml THF added. After 12 h at rt, NH_4Cl-solution was added and the solution extracted three times with 20 ml DCM. The combined organic layers were dried over $MgSO_4$, filtered and the solvent was removed under reduced pressure. After purification by column chromatography on silica (SiO_2, PE:EA, 10:1), **44** (4.08 g, 14.8 mmol, 82%) was obtained as a yellow oil.

R_f (PE:EA, 3:1) = 0.36; IR (Film): $\tilde{\nu}$ = 3421 cm^{-1}, 2953, 2918, 2860, 2836, 1613, 1586, 1513, 1361, 1302, 1249, 1174, 1097, 1034, 818, 788; ^1H NMR ($CDCl_3$, 250 MHz): δ = 2.07–2.17 (m, 2 H), 2.27 (d, ^4J = 0.9 Hz, 3 H), 3.06 (s, 1 H), 3.57–3.74 (m, 2 H), 3.80 (s, 3 H), 4.46 (s, 2 H), 4.86 (t, ^3J = 6.2 Hz, 1 H), 5.89 (dq, ^3J = 3.1 Hz, ^4J = 0.9 Hz, 1 H), 6.09 (d, ^3J = 3.1 Hz, 1 H), 6.84–6.92 (m, 2 H), 7.22–7.32 (m, 2 H); ^{13}C NMR ($CDCl_3$, 63 MHz): δ = 13.68 (q), 35.19 (t), 55.42 (q), 67.02 (d), 68.02 (t), 73.08 (t), 106.13 (d), 106.85 (d), 114.09 (d, 2 C), 129.43 (d, 2 C), 130.22 (s), 151.71 (s), 154.82 (s), 159.45 (s); MS (EI (+)): *m/z* (%): 276 $[M]^+$ (1), 259 (100), 155 (17), 121

(24); $C_{16}H_{20}O_4$ (276.33): calcd. C 69.54, H 7.30; found C 69.96, H 7.38; HRMS (EI (+)): $C_{16}H_{20}O_4$: calcd. 276.1367; found 276.1362.

2-{3-[(4-Methoxybenzyl)oxy]-1-(prop-2-yn-1-yloxy)propyl}-5-methylfuran (**45**)

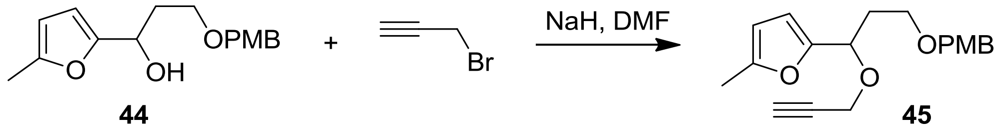

Compound **44** (1.13 g, 4.09 mmol) was dissolved in 10 ml DMF and NaH (124 mg, 4.91 mmol, 95%) added at 0 °C. After 30 min, propargyl bromide (684 µl, 6.14 mmol, 80% in toluene) was added and the solution stirred 4 h. Water was added and the solution extracted with Et_2O. The combined organic layers were dried over $MgSO_4$, filtered and the solvent was removed under reduced pressure. After purification by column chromatography on silica (SiO_2, PE:EA, 25:1), **45** (1.16 g, 3.70 mmol, 90%) was obtained as a yellow oil.

R_f (PE:EA, 3:1) = 0.70; IR (Film): $\tilde{\nu}$ = 3421 cm^{-1}, 3288, 3261, 3196, 3954, 3932, 2860, 2171, 1613, 1586, 1560, 1514, 1442, 1361, 1326, 1302, 1248, 1174, 1098, 1079, 1035, 818, 790, ^{1}H NMR ($CDCl_3$, 250 MHz): δ = 2.00–2.31 (m, 2 H), 2.27 (d, ^4J = 1.0 Hz, 3 H), 2.38 (t, ^4J = 2.4 Hz, 1 H), 3.41–3.51 (m, 1 H), 3.56-3.65 (m, 1 H), 3.80 (s, 3 H), 3.95 (dd, ^2J = 15.6 Hz, ^4J = 2.4 Hz, 1 H), 4.13 (dd, ^2J = 15.6 Hz, ^4J = 2.4 Hz, 1 H), 4.40 (d, ^2J = 11.5 Hz, 1 H), 4.46 (d, ^2J = 11.5 Hz, 1 H), 4.65 (dd, ^3J = 8.1 Hz, ^3J = 6.0 Hz, 1 H), 5.89 (dq, ^3J = 3.1 Hz, ^4J = 1.0 Hz, 1 H), 6.18 (d, ^3J = 3.1 Hz, 1 H), 6.84–6.91 (m, 2 H), 7.22–7.31 (m, 2 H); ^{13}C NMR ($CDCl_3$, 63 MHz): δ = 13.79 (q), 34.25 (t), 55.40 (q), 55.52 (t), 66.44 (t), 70.68 (d), 72.73 (t), 74.16 (s), 80.11 (d),

106.01(d), 110.18 (d), 113.85 (d, 2 C), 129.36 (d, 2 C), 130.77 (s), 151.07 (s), 152.58 (s), 159.23 (s); MS (EI (+)): *m/z* (%): 314 (4) $[M]^+$, 193 (85), 149 (21), 122 (81), 121 (100), 109 (22), 73 (19).; $C_{19}H_{22}O_4$ (314.38): calcd. C 72.59, H 7.05; found C 72.51, H 7.08.

3-(5-Methylfuran-2-yl)-3-(prop-2-yn-1-yloxy)propan-1-ol (**39**)

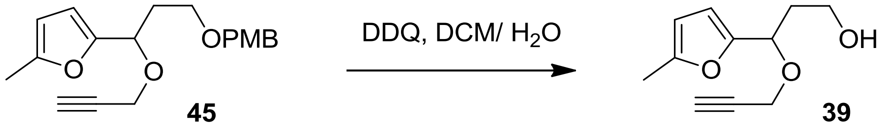

Compound **45** (1.39 g, 4.42 mmol) was dissolved in 50 ml DCM and 10 ml H_2O. DDQ (1.20 g, 5.30 mmol) was added and the reaction mixture stirred for 2 h at rt. After the addition of sat. $NaHCO_3$ solution, the aqueous phase was extracted with DCM. The combined organic layers were dried over $MgSO_4$, filtered and the solvent was removed under reduced pressure. After purification by column chromatography on silica (SiO_2, PE:EA, 10:1), **39** (562 mg, 2.89 mmol, 65%) was obtained as a yellow oil.

R_f (PE:EA, 5:1) = 0.11; IR (neat): $\tilde{\nu}$ = 3291 cm^{-1}, 2953, 2923, 2883, 1560, 1439, 1355, 1219, 1050, 1019, 963, 939, 787, 648; ^1H NMR (CD_3CN, 500 MHz): δ = 1.85-1.92 (m, 1 H), 2.00-2.07 (m, 1 H), 2.24 (s, 3 H), 2.61 (t, ^3J = 5.3 Hz, 1 H), 2.65 (t, ^4J = 2.4 Hz, 1 H), 3.44-3.50 (m, 1 H), 3.53-3.59 (m, 1 H), 3.92 (dd, ^2J = 15.8 Hz, ^4J = 2.4 Hz 1 H), 4.05 (dd, ^2J = 15.8 Hz, ^4J = 2.4 Hz 1 H), 4.56 (dd, ^3J = 7.9 Hz, ^3J = 6.1 Hz, 1 H), 5.96-5.97 (m, 1 H), 6.32 (d, ^3J = 3.2 Hz, 1 H); ^{13}C NMR (CD_3CN, 126 MHz): δ =

13.60 (q), 37.58 (t), 55.94 (t), 59.18 (t), 70.25 (d), 75.38 (d), 81.00 (s), 106.97 (d), 110.99 (d), 152.35 (s), 153.38 (s); MS (ESI (+)): *m/z* (%): 217 (100). HRMS (ESI (+)): $C_{11}H_{14}NaO_3$: calcd. 217.0835; found 217.0834.

1-{2-[(4-Methoxybenzyl)oxy]ethyl}-5-methyl-1,3-dihydro-2-benzofuran-4-ol (**46**)

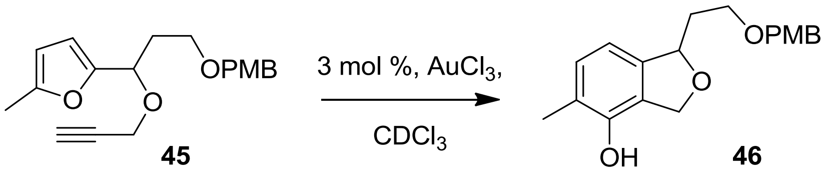

Compound **45** (100 mg, 318 µmol) and $AuCl_3$ (2.89 mg, 9.54 µmol) in 3 ml $CDCl_3$ were allowed to react for 10 min. After evaporation of the solvent, the crude product was purified by column chromatography on silica (SiO_2, PE:EA, 10:1) to give **46** (89.7 mg, 285 µmol, 90 %) as a colorless oil.

R_f (PE:EA = 3:1) = 0.31; IR (Film): $\tilde{\nu}$ = 3324 cm^{-1}, 2918, 2858, 1612, 1513, 1463, 1248, 1093, 1075, 1034, 634, 548, 532, 508; ^{1}H NMR ($CDCl_3$, 250 MHz): δ = 1.75–2.27 (m, 2 H), 2.41 (s, 3 H), 3.71–3.93 (m, 2 H), 3.97 (s, 3 H), 4.59 (d, ^2J = 11.8 Hz, 1 H), 4.65 (d, ^2J = 11.8 Hz, 1 H), 4.88 (s, 1 H), 5.20 (dd, ^2J = 12.0 Hz, ^4J = 1.6 Hz, 1 H), 5.20 (dd, ^2J = 12.0 Hz, ^4J = 2.5 Hz, 1 H), 5.47– 5.57 (m, 1 H), 6.81 (d, ^3J = 7.5 Hz, 1 H), 6.90–7.08 (m, 2 H), 7.19 (d, ^3J = 7.5 Hz, 1 H), 7.39–7.46 (m, 2 H); ^{13}C NMR ($CDCl_3$, 63 MHz): δ = 15.18 (q), 36.79 (t), 55.43 (q), 66.90 (t), 70.56 (t), 73.00 (t), 81.78 (d), 113.40 (d), 113.96 (d, 2 C), 121.94 (s), 125.40 (s), 129.49 (d, 2 C), 130.63 (d), 130.68 (s), 142.45 (s), 148.16 (s), 159.26 (s); MS (CI (+)): *m/z* (%): 313 (44) [M-

H]$^+$, 193 (100), 149 (23), 121 (63); HRMS (CI (+)): $C_{19}H_{21}O_4$ ([M-H])$^+$: calcd. 313.1440; found 313.1421.

1-(2-Hydroxyethyl)-5-methyl-1,3-dihydro-2-benzofuran-4-ol (**40**)

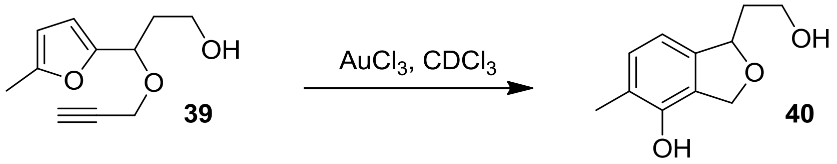

200 mg (1.03 mmol) **39** and 15.6 mg (51.5 μmol) $AuCl_3$ were converted in 3 ml $CDCl_3$ for 30 min at room temperature. After evaporation, the crude product was purified by column chromatography on silica (SiO_2, PE:EA, 2:1). 133 mg (685 µmol, 66%) **40** was gained as a colourless solid.

Mp.: 142 °C; R_f (PE:EA, 1:1) = 0.13. IR (neat): $\tilde{\nu}$ = 3345 cm^{-1}, 3213, 2863, 1625, 1459, 1437, 1382, 1347, 1319, 1292, 1234, 1047, 1025, 1012, 898, 849, 807, 645, 617; ^{1}H NMR (MeOD, 500 MHz): δ = 1.82-1.89 (m, 1 H), 2.08 (dtd, ^2J = 14.0 Hz, ^3J = 7.4 Hz, ^3J = 3.4 Hz, 1 H), 2.23 (s, 3 H), 3.70-3.80 (m, 2 H), 4.98 (d, ^2J = 12.0 Hz, 1 H), 5.07 (dd, ^2J = 12.0 Hz, J = 2.5 Hz, 1 H), 5.28-5.31 (m, 1 H), 6.63 (d, ^3J = 7.6 Hz, 1 H), 7.03 (d, ^3J = 7.6 Hz, 1 H);* ^{13}C NMR (MeOD, 126 MHz): δ = 16.70 (q), 41.10 (t), 60.69 (t), 72.45 (t), 83.77 (d), 113.88 (d), 125.73 (s), 127.07 (s), 132.47 (d), 143.46 (s), 151.21 (s); MS (EI(+)): *m/z* (%): 194 (23) [M]$^+$, 149 (100), 121 (10), 91 (10), 77 (8). HRMS (EI (+)):$C_{11}H_{14}O_3$: calcd. 194.0943; found 194.0940. * OH-groups not detected due to H/D exchange
